# Lymph node targeted multi-epitope subunit vaccine promotes effective immunity to EBV in HLA-expressing mice

**DOI:** 10.1038/s41467-023-39770-1

**Published:** 2023-08-08

**Authors:** Vijayendra Dasari, Lisa K. McNeil, Kirrilee Beckett, Matthew Solomon, George Ambalathingal, T. Le Thuy, Archana Panikkar, Caitlyn Smith, Martin P. Steinbuck, Aniela Jakubowski, Lochana M. Seenappa, Erica Palmer, Jeff Zhang, Christopher M. Haqq, Peter C. DeMuth, Rajiv Khanna

**Affiliations:** 1 QIMR Centre for Immunotherapy and Vaccine Development, Tumour Immunology Laboratory, Infection and Inflammation Program, Berghofer Medical Research Institute, Brisbane, Australia; 2 Elicio Therapeutics, Inc, Boston, MA USA

**Keywords:** Protein vaccines, Herpes virus, Viral infection, Infection

## Abstract

The recent emergence of a causal link between Epstein-Barr virus (EBV) and multiple sclerosis has generated considerable interest in the development of an effective vaccine against EBV. Here we describe a vaccine formulation based on a lymph node targeting Amphiphile vaccine adjuvant, Amphiphile-CpG, admixed with EBV gp350 glycoprotein and an engineered EBV polyepitope protein that includes 20 CD8^+^ T cell epitopes from EBV latent and lytic antigens. Potent gp350-specific IgG responses are induced in mice with titers >100,000 in Amphiphile-CpG vaccinated mice. Immunization including Amphiphile-CpG also induces high frequencies of polyfunctional gp350-specific CD4^+^ T cells and EBV-specific CD8^+^ T cells that are 2-fold greater than soluble CpG and are maintained for >7 months post immunization. This combination of broad humoral and cellular immunity against multiple viral determinants is likely to provide better protection against primary infection and control of latently infected B cells leading to protection against the development of EBV-associated diseases.

## Abstract (web summary)

There is a clinical need for effective and efficacious vaccines for Epstein-Barr virus (EBV) that induce substantive and protective immunity. Here the authors use a combined lymph-node targeted adjuvant and subunit vaccine against EBV and show the induction and effectiveness in a human leukocyte antigen expressing murine model.’

## Introduction

Epstein-Barr virus (EBV) is a gamma herpesvirus and is a ubiquitous human pathogen, infecting at least 95% of the world’s adult population. During primary infection EBV is generally transmitted through saliva and infects resting B cells or epithelial cells in the oropharynx and then triggers transcriptional programming of B cells to establish life-long viral latency^1^. Primary EBV infection in children is asymptomatic or mildly symptomatic. Further, while acquisition of EBV infection in adolescents or young adults is often asymptomatic, development of symptomatic infectious mononucleosis (IM) occurs in a subset of patients^2^. In addition to significant primary infection-associated morbidity, IM is considered to be a major risk factor for the future development of multiple EBV-associated diseases including multiple sclerosis (MS) and Hodgkin lymphoma^3,4^. In addition, EBV is considered the primary etiological agent associated with multiple lymphoid and epithelial cancers with about 200,000 new EBV-associated cancers diagnosed worldwide each year^5,6^.

Several prophylactic and therapeutic approaches are in various stages of development, but no medical intervention for EBV infection has been licensed to date^1,7^. Recently, Moderna and the NIH launched two separate Phase I clinical trials to study safety and immunogenicity of EBV vaccination in 18-30 young healthy adults: clinical evaluation of mRNA vaccination by Moderna (mRNA–1189; NCT05164094) targets four glycoprotein antigens (gH, gL, gp42, and gp220), while the NIH study (NCT04645147) will evaluate an investigational multimeric EBV gp350-Ferritin nanoparticle vaccine with a saponin-based Matrix-M adjuvant^8–11^. Additional assessments of a multimeric EBV gp350 vaccine are ongoing^12^. In EBV-infected individuals, serum antibodies directed against EBV-encoded glycoproteins (e.g. glycoprotein 350, gp350) are able to neutralize EBV to prevent infection of B cells and epithelial cells^8,13–15^. Human Phase II clinical trials with gp350/AS04 vaccination demonstrated clinical efficacy in preventing the development of acute IM but did not prevent EBV infection^16^. Although preexisting neutralizing antibodies provide a first line of defense against acute viral infection, it is now well established that effective long-term control of latently infected B cells is critically dependent on T cell-mediated immunity. Therefore, induction of potent and durable T cell immunity through vaccination may prevent establishment or progression of latent EBV infection and the subsequent development of etiologically related autoimmune diseases and cancers.

Based on these observations, optimal EBV vaccines designed to control primary and latent infection will need to induce both humoral and cellular immune responses targeted to viral glycoproteins, lytic and latent antigens including EBV nuclear antigens and latent membrane proteins. Subunit vaccination is an attractive strategy for this purpose, however, full-length EBV latent proteins can trigger oncogenesis by blocking apoptosis, promoting genomic instability, and supporting uncontrolled cell proliferation which limits their use as vaccine immunogens^17,18^. Moreover, polyvalent strategies requiring manufacturing and co-formulation of numerous protein immunogens required to induce the necessary breadth of immunity may not be feasible due to challenging cost and complexity of manufacturing. Accordingly, we have developed a strategy to specifically target multiple latent and lytic EBV protein epitopes which overcomes these factors limiting their use in a vaccine formulation. Extensive mapping of human CD8^+^ T cell epitopes from EBV antigens by multiple groups^19–30^ has allowed us to design a polyepitope vaccine immunogen (EBVpoly) that incorporates multiple CD8^+^ T cell epitopes into an engineered protein immunogen. Here, individual HLA-restricted, highly conserved antigenic peptide sequences identified from a variety of EBV antigens are included in series and separated by proteolytic cleavage sites creating a structure resembling “beads on a string”^31–34^. Inclusion of epitopes from both latent and lytic antigens is intended to promote generation of T cell responses directed to different phases of the EBV life cycle. In addition, peptide epitopes restricted through multiple common HLA class I alleles were included to allow broad worldwide coverage across multiple ethnic groups. However, for vaccinated individuals the potential breadth of T cell responses generated with EBVpoly will be dependent on the specific HLA alleles expressed. Preclinical studies have shown that this polyepitope vaccine strategy is highly effective in inducing anti-viral T cell immunity targeting EBV and CMV^32,33,35^. Co-formulation of EBVpoly with whole recombinant gp350 protein presents an opportunity to generate EBV-neutralizing humoral responses in concert with cellular immunity to enhance prophylactic activity alongside persistent control of EBV latency.

In addition to immunogen selection, incorporation of immunomodulatory adjuvants in vaccine design can determine the strength and character of the resulting immune response. Molecular adjuvants, including TLR agonists, are attractive options for use in subunit vaccines given their potential for simple co-formulation and potent immunomodulation in support of adaptive immunity. However, rapid capillary absorption of low molecular weight adjuvants (<20 kDa) at the injection site leads to limited accumulation in draining lymph nodes where adaptive immune responses are orchestrated. In contrast, larger macromolecules and proteins, including many subunit immunogens, are restricted from transit across the vascular endothelium by multiple size-dependent anatomical structures, leading to preferential drainage from tissue through afferent lymphatics. To address this challenge, we have utilized an Amphiphile (AMP)-modified CpG DNA adjuvant (AMP-CpG) designed to enhance lymph node delivery and accumulation. Here, diacyl-lipid conjugation to TLR-9 stimulatory CpG DNA facilitates association with endogenous tissue albumin (~65 kDa) at the injection site to achieve specific targeting to the draining lymph nodes where early activation of the immune response is initiated^36–40^. Co-administration with protein subunit immunogens of relatively large molecular size therefore allows for concerted accumulation of antigen and adjuvant in draining lymph nodes to promote potent immunity.

Using EBV gp350 and EBVpoly in combination with AMP-CpG, we have designed a vaccine formulation to stimulate potent and durable humoral and cellular immunity and assessed its immunogenicity in multiple HLA-expressing mouse models. The data show that this strategy elicits robust and persistent EBV-specific neutralizing antibodies and polyfunctional antigen-specific CD4^+^ and CD8^+^ T cell responses.

## Results

### EBV vaccine design

**Fig. 1:**
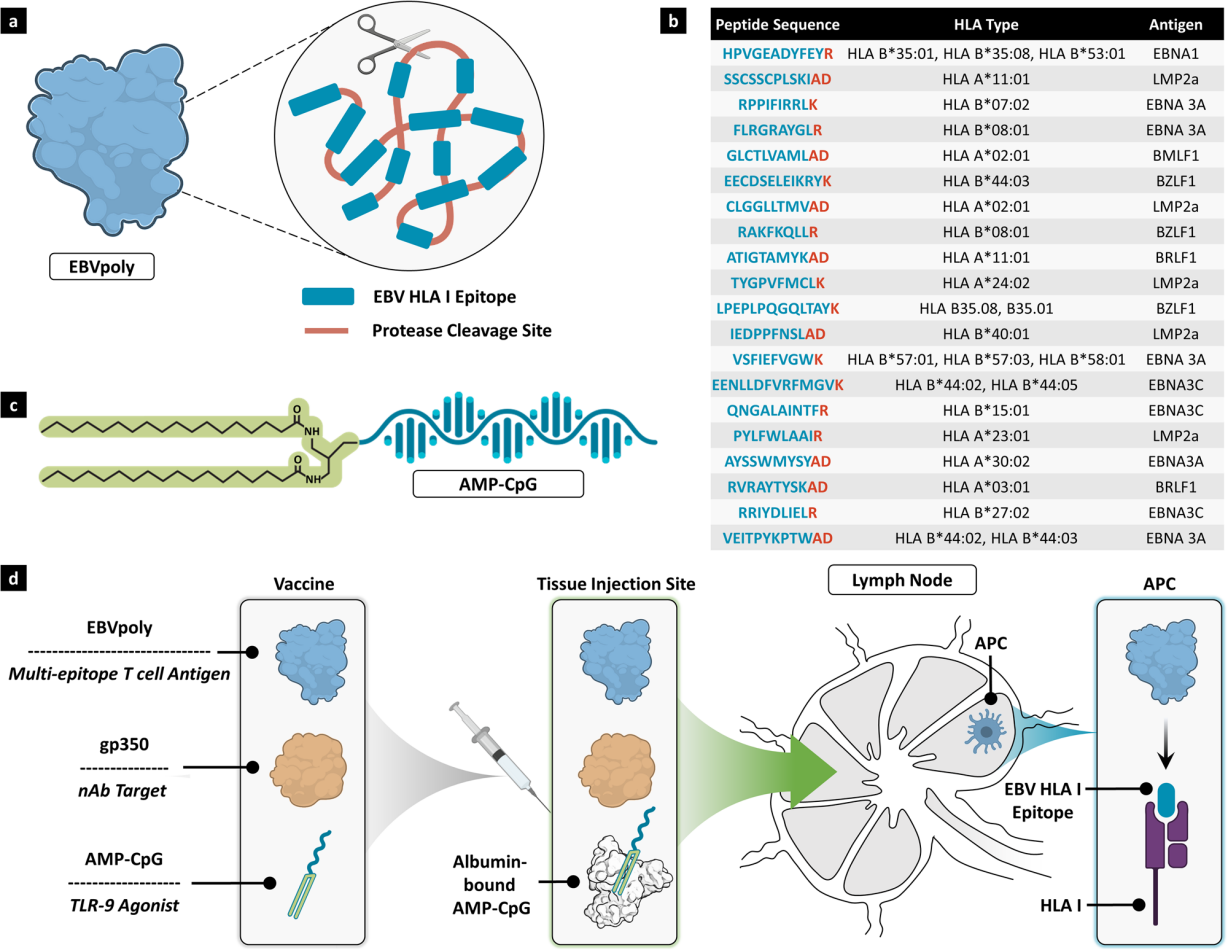
Design of a lymph node-targeted subunit vaccine for EBV. **a** Schematic representation of EBVpoly protein, showing the “beads on a string” structure. EBVpoly is a polyprotein containing 20 CD8^+^ T cell epitopes from eight EBV antigens. The peptide sequence, HLA type and source antigen for each of the epitopes are listed in **b** with the proteasome liberation sequence highlighted in red. **c** Schematic representation of the adjuvant, AMP-CpG. **d** Vaccine design and mechanism of vaccination including delivery to the lymph nodes following subcutaneous injection. EBVpoly is an engineered multi-epitope protein immunogen consisting of multiple HLA-restricted T cell target epitopes; gp350 is a predominant viral target for neutralizing antibody responses; AMP-CpG is a lymph node-targeted TLR-9 agonist. AMP-CpG binds to endogenous albumin at the injection site and albumin chaperones AMP-CpG into the lymph nodes in concert with passive transport of EBVpoly and gp350. Lymph node APCs internalize and process EBVpoly and the individual epitopes are presented on HLA Class I to CD8^+^ T cells. Created with BioRender.com.

To design a vaccine for EBV capable of inducing both humoral and T cell responses against multiple viral antigens, we employed an engineered immunogen design strategy together with an optimal lymph node-targeted adjuvant approach to ensure concerted delivery of T and B cell epitopes with lymphatic immune activation. We first designed EBV polyepitope protein, EBVpoly, an engineered recombinant polyprotein with 20 CD8^+^ T cell epitopes from eight different lytic and latent EBV antigens, linked together to form a “beads on a string” structure (Fig. 1a, b and Supplementary Fig S1). The EBV epitopes were selected to target broad HLA coverage and multiple antigens representative of all phases of the EBV life cycle. The HLA Class-I restricted A and B coverage of EBVpoly is 92% for the world population, with 94% coverage in the United States^41^ and average potential recognition of 2.66 epitopes by immunized individuals predicted through HLA haplotype analysis^42^. To facilitate effective epitope processing for HLA-presentation, the carboxy terminus of each epitope was joined by a proteasome liberation amino acid sequence (AD or K or R). These proteasomal liberation sequences were designed to improve the immunogenicity of the EBV CD8^+^ T cell epitopes by facilitating proteasomal processing of the polyepitope protein after uptake by antigen presenting cells (APCs). The EBV gp350 protein was also included in the vaccine formulation as a known target for virus neutralization as well as CD8^+^ and CD4^+^ T cell responses. To promote robust lymphatic immune activation in concert with immunogen delivery, a lymph node-targeted AMP adjuvant, AMP-CpG, was included (Fig. 1c). After peripheral administration, AMP-CpG (consisting of a diacyl lipid conjugated to TLR-9 agonistic CpG DNA) non-covalently associates with endogenous tissue albumin which is efficiently transported via afferent lymphatics to accumulate in draining lymph nodes^38^. While prior studies have shown the small molecular size of CpG DNA necessitates AMP-modification to achieve lymph node targeting, the larger size of EBVpoly and gp350 proteins predicts their effective transit from subcutaneous tissue into lymph^43^. Once in the lymph nodes, the vaccine components are designed to accumulate in APCs where AMP-CpG can stimulate immune activation^38,44^ while EBVpoly is processed to yield distinct epitopes for MHC Class I presentation and gp350 is available for T and B cell recognition (Fig. 1d).

### EBVpoly effectively stimulates effector responses in human T cells

**Fig. 2:**
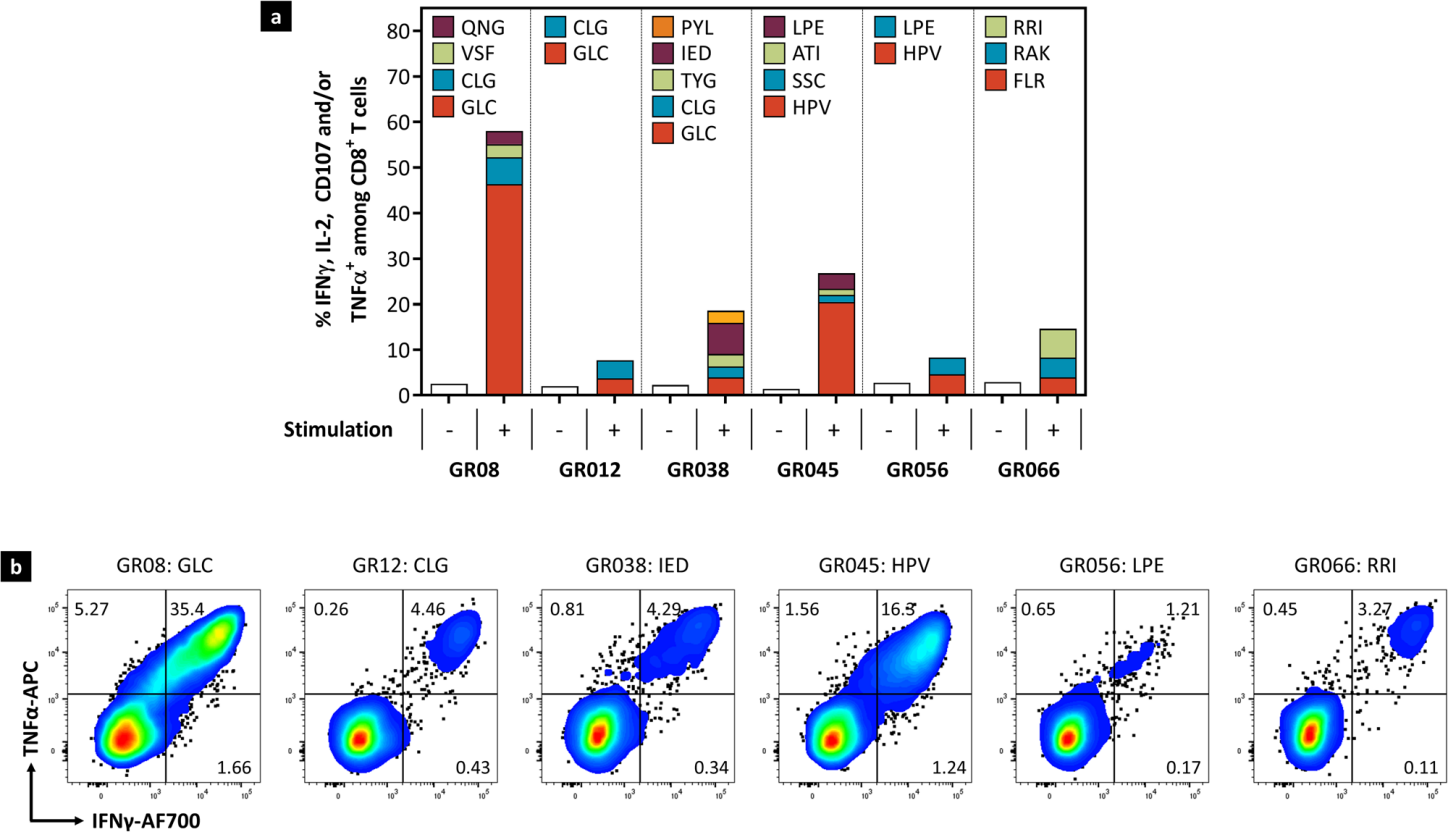
EBVpoly stimulation of human PBMCs expands EBV-specific CD8^+^ T cells. **a** PBMCs from healthy EBV seropositive patients were stimulated with EBVpoly for 1 hour, then expanded for 14 days in the presence of IL-2. Expanded PBMCS were re-stimulated with the individual antigen peptides from EBVpoly in an ICS assay. Shown are frequencies of IFNγ, IL-2, TNFα, and/or CD107^+^ CD8^+^ T cells. White bars indicate no peptide stimulation. Colored bars represent responses to each annotated peptide designated based on the first three amino acid residues of the cognate epitope. **b** Shown are representative flow cytometry dot plots of IFNγ vs TNFα for each donor demonstrating responses to the designated peptide re-stimulation. Data are representative of one experiment. Source data are provided as a Source Data file.

To confirm that EBVpoly can be processed by APCs and presented on HLA Class I molecules to activate EBV-specific CD8^+^ T cell responses in humans, peripheral blood mononuclear cells (PBMCs) from six healthy EBV seropositive donors, HLA-matched for the epitopes expressed in EBVpoly, were pre-incubated with EBVpoly and then cultured with IL-2 for 14 days to expand pre-existing memory CD8^+^ T cells. The expansion of the EBV-specific T cells was then assessed by intracellular cytokine staining (ICS) assay. EBVpoly induced simultaneous expansion of functional EBV-specific CD8^+^ T cells to multiple EBV epitopes in all six donors evaluated (Fig. 2a). These expanded T cells showed polyfunctional effector function following stimulation with EBV peptide epitopes as indicated by the expression of both IFNγ and TNFα (Fig. 2b). These data confirm that EBVpoly can be efficiently processed by APCs, yielding epitopes effectively presented on MHC and recognized by cognate human CD8^+^ T cells to promote activation and effector function.

### AMP-CpG is associated with improved accumulation of EBVpoly alongside persistent inflammation of local and distal lymph nodes

**Fig. 3:**
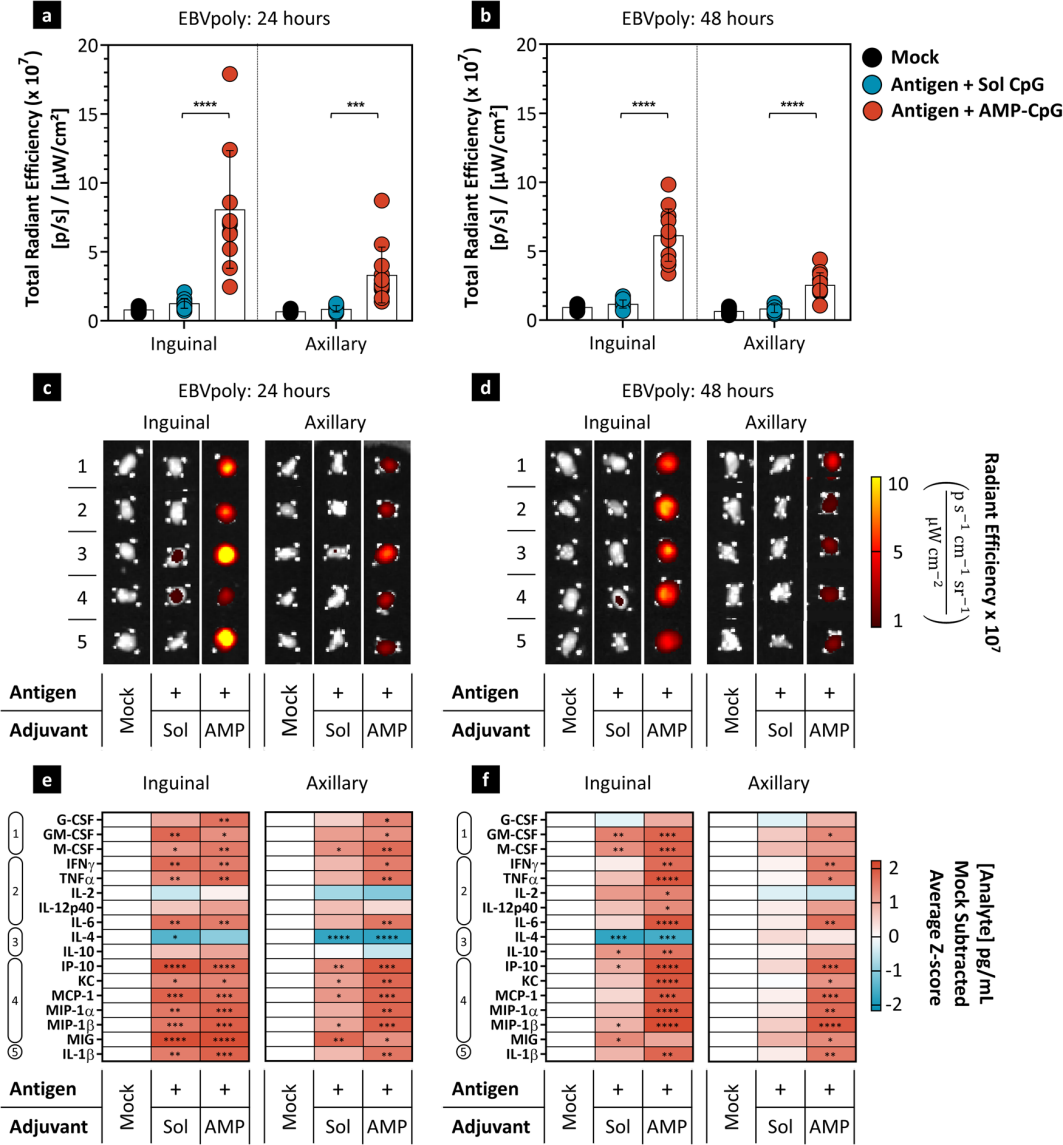
AMP-CpG enhances delivery of EBVpoly to the lymph node alongside comprehensive immune activation. C57Bl/6J mice were immunized with 8 µg EBVpoly-AF594 and 10 µg gp350-AF647 admixed with 1.2 nmol soluble or AMP-CpG (*n* = 6 mice per group, 2 lymph nodes per mouse). **a**–**d** Quantification of total radiant efficiency in inguinal and axillary lymph nodes analyzed ex vivo by IVIS at **a** 24 and **b** 48 h post primer dose. Mock treatments represent fluorescence-negative controls collected from mice receiving equivalent amounts of AMP-CpG and unlabeled antigens. **c**, **d** IVIS images of five representative lymph nodes in **a** and **b**. **e**, **f** Quantification of cytokine concentrations in lymph nodes from **a** and **b** by Luminex. Depicted are mock-subtracted average *Z*-scores of protein analyte concentrations in lymph nodes. Cytokines are clustered into functional groups: (1) growth factors, (2) Th1/inflammatory cytokines, (3) Th2/regulatory cytokines, (4) chemokines, and (5) inflammasome-associated cytokines. Mock-treated animals were administered vehicle alone. Data are representative of one experiment. Values depicted are mean ± standard deviation. *p < 0.05; **p < 0.01; ***p < 0.001; ****p < 0.0001, **e**, **f** ANOVA, Dunnett multiple comparisons test, FDR 2-stage, step-up Benjamini, Krieger, and Yukatiele analysis, *Q* = 5%. Source data are provided as a Source Data file.

Transit of molecules to the lymph nodes from a peripheral injection site is known to correlate with molecular size^38,39^ and the efficiency of lymphatic drainage can be further modulated in response to local adjuvant-mediated inflammatory activity^45^. To investigate the efficiency of lymph node accumulation of EBVpoly and gp350, we administered fluorescently labeled proteins in mice together with soluble CpG or AMP-CpG and analyzed tissue-associated fluorescent signal in whole lymph nodes by IVIS imaging. EBVpoly (~25 kDa) accumulation in local and distal lymph nodes was significantly enhanced by co-administration with AMP-CpG relative to soluble CpG 24 and 48 hours after injection (Fig. 3a–d**)**. In soluble CpG immunized animals, EBVpoly was only detectable ~1.5-fold above mock injected control levels in inguinal lymph nodes 24 h following administration. Co-administration with AMP-CpG induced persistent accumulation in both inguinal and axillary nodes as much as 9.8-fold above background at 24 and 48 h suggesting a potential mechanism for AMP-CpG mediated enhancement of lymph node drainage and/or retention for small protein immunogens. In contrast, gp350 (~350 kDa) was observed in both local inguinal and distal axillary lymph nodes at 24 and 48 h with similar levels of accumulation after soluble and AMP-CpG adjuvanted vaccination (Supplementary Fig. S2).

Parallel observation of proteomic signatures of inflammation in draining lymph nodes was conducted through multiplexed assessment of a panel of inflammatory or immunomodulatory proteins including (1) growth factors, (2) Th1-associated pro-inflammatory cytokines, (3) Th-2 and regulatory cytokines, (4) chemokines, and (5) inflammasome-associated cytokines. Analysis of the proteomic milieu in inguinal lymph nodes at 24 h revealed a similar profile of upregulation in animals immunized with soluble or AMP-CpG, characterized by comprehensive elevation of nearly all analytes evaluated (Fig. 3e and Supplementary Fig. S3). For AMP-CpG, this profile was largely consistent with that observed in distal axillary lymph nodes, while soluble CpG did not induce significant upregulation of a majority of analytes in distal axillary lymph nodes. Inflammatory proteomic responses induced by soluble CpG largely reverted to baseline in inguinal lymph nodes at 48 h, consistent with a complete attenuation of response in distal axillary lymph nodes at this time point (Fig. 3f). However, AMP-CpG-induced inflammatory protein signatures were largely maintained in local and distal lymph nodes at 48 h suggesting a more durable and comprehensive induction of acute inflammation associated with improved lymph node targeting by AMP-mediated delivery.

### Cellular immune response

**Fig. 4:**
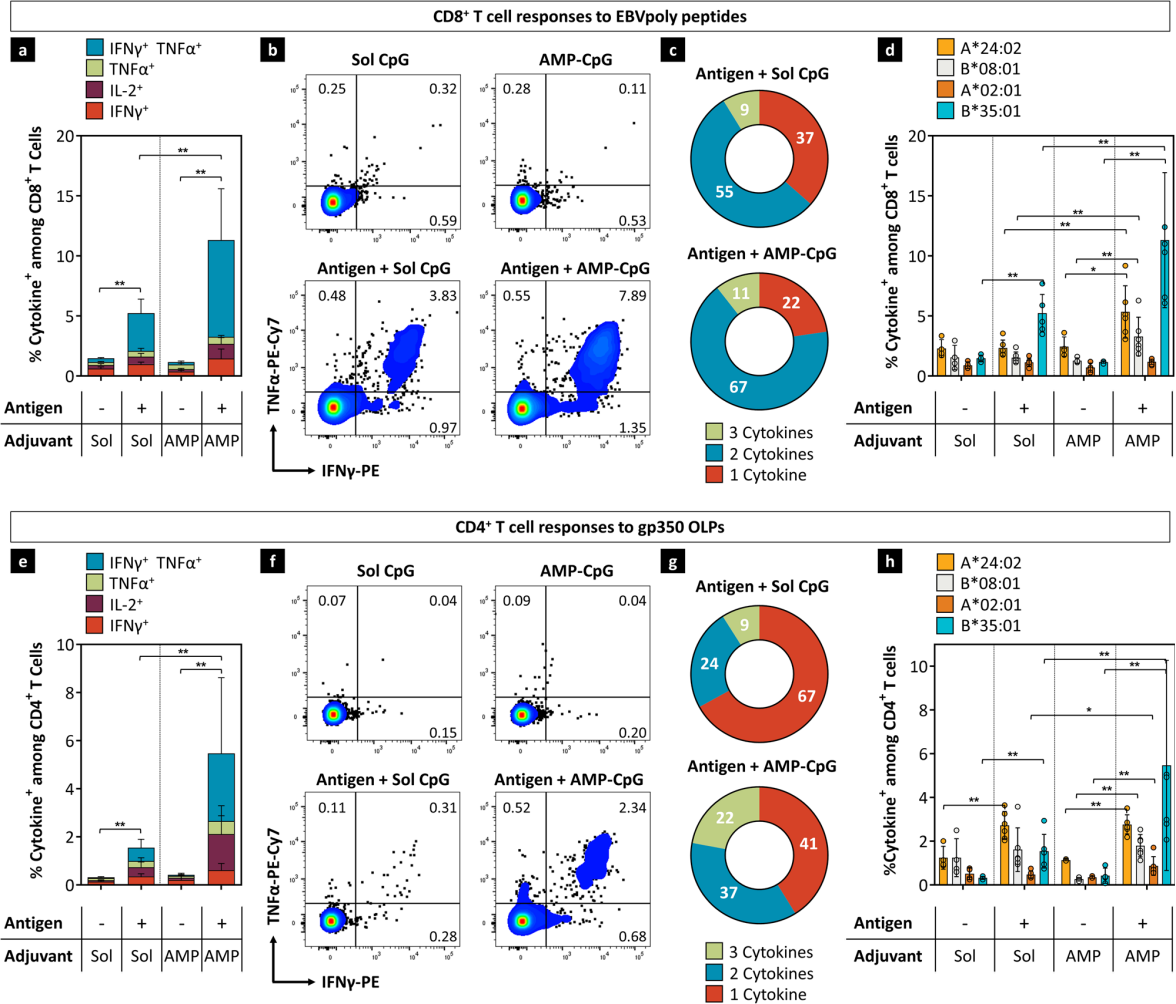
Vaccination with AMP-CpG induces robust polyfunctional EBV-specific T cell responses in splenocytes. **a**–**h** HLA-B*35:01 transgenic mice (*n* = 6 per vaccine group and *n* = 4 per control group) were immunized at weeks 0, 3 and 6 with 40 μg EBVpoly and 10 μg gp350 proteins admixed with 1.2 nmol soluble or AMP-CpG and T cell responses were analyzed at week 7. Control groups were immunized with soluble CpG or AMP-CpG alone. Splenocytes were stimulated with **a**–**d** EBV CD8^+^ T cell peptides or **e**–**h** gp350 OLPs in an ICS assay. **a**, **e** Shown are frequencies of IFNγ, IL-2, TNFα, and IFNγ TNFα double positive CD8^+^ or CD4^+^ T cells, with **b**, **f** corresponding representative dot plots. **c**, **g** Pie chart representations of the functional T cell profiles. Pies represent the capacity of T cells to secrete any (1, 2, or 3) of the three cytokines IFNγ, TNFα, and IL-2. **d**, **h** Frequencies of cytokine^+^ **d** CD8^+^ T cells and **h** CD4^+^ T cells in splenocytes to indicated HLA transgenic mice. Data are representative of one experiment. Values depicted are mean ± standard deviation. *p < 0.05; **p < 0.01 by two-sided Mann-Whitney test applied to cytokine^+^ T cell frequencies. If statistics are not indicated, then the comparison was not significant. The exact *p*-values are as follows. **a** Soluble vaccine to soluble CpG-0.0095, AMP vaccine to soluble vaccine-0.0087, and AMP vaccine to AMP CpG-0.0095. **d** Soluble vaccine to soluble CpG for B*35:01-0.0095, AMP vaccine to soluble vaccine for A*24:01-0.0043, AMP vaccine to AMP-CpG for A*24:01-0.0190, AMP vaccine to AMP-CpG for B*35:01-0.0095, and AMP vaccine to soluble vaccine for B*35:01-0.0087. **e** Soluble vaccine to soluble CpG-0.0095, AMP vaccine to soluble vaccine-0.0087, and AMP vaccine to AMP CpG-0.0095. **h** Soluble vaccine to soluble CpG for A*24:01-0.0095, soluble vaccine to soluble CpG for B*35:01-0.0095, AMP vaccine to soluble vaccine for A*02:01-0.0152, AMP vaccine to soluble vaccine for B*35:01-0.0087, AMP vaccine to AMP-CpG for A*24:01-0.0095, AMP vaccine to AMP-CpG for B*35:01-0.0095, AMP vaccine to AMP-CpG for A*02:01-0.0095, and AMP vaccine to AMP-CpG for B*08:01-0.0095. Source data are provided as a Source Data file.

To evaluate the strength and character of EBV-specific T cells and further establish the effect of a lymph node-targeted adjuvant on EBV-specific cellular immunity, we compared vaccination with AMP-CpG to soluble CpG in HLA transgenic mice. HLA-B*35:01 mice were primed and then boosted twice, each three weeks apart with gp350 and EBVpoly admixed with dose-matched AMP-CpG or soluble CpG. EBV-antigen-specific cytokine-producing T cells in splenocytes were evaluated directly ex vivo at week 7, 1 week post second boost. Adjuvant only control immunized mice did not induce detectable T cell responses, while mice immunized with AMP-CpG had significantly higher EBVpoly-specific CD8^+^ cytokine^+^ T cells compared to mice dosed with soluble CpG (Fig. 4a–c). Approximately 11% of CD8^+^ T cells produced either IFNγ, TNFα, IL-2, or both IFNγ and TNFα in AMP-CpG immunized mice, with 78% exhibiting polyfunctional secretion of two and/or three cytokines. By comparison, immunization with soluble CpG induced only 5% CD8^+^ T cell responses. CD8^+^ T cell responses after AMP-CpG immunization were also observed for other HLA transgenic mice, A*24:02 and B*08:01 at higher levels than soluble CpG, although frequencies of cytokine-positive cells were lower than observed in B*35:01 mice (Fig. 4d). Cytokine-producing CD4^+^ T cells in splenocytes stimulated ex vivo with gp350 overlapping peptides (OLPs) were also evaluated. AMP-CpG induced robust gp350-specific CD4^+^ cytokine^+^ T cells, ~3-fold higher than observed in soluble CpG immunized comparator mice (Fig. 4e, f) with correlated improvements in polyfunctional cytokine secretion (59% compared to 33%, respectively, Fig. 4g). EBV gp350-specific CD4^+^ T cell responses were also induced in the other HLA transgenic mice, albeit at lower frequencies (Fig. 4h). AMP-CpG immunization also induced three-fold higher gp350-specific CD8^+^ T cells than observed in adjuvant-only control-treated animals with ~1.7% cytokine^+^ cells at week 7 (Supplementary Fig. S4). Further, EBVpoly-specific CD8^+^ T cell responses and gp350-specific CD4^+^ T cells responses were also observed in draining inguinal and axillary lymph nodes (Supplementary Fig. S5).

To enrich for EBV-specific memory T cells, splenocytes from the immunized mice were stimulated with EBVpoly CD8^+^ T cell peptides or gp350 OLPs for 10 days, then assessed for the frequency of antigen-specific cytokine-producing T cells. Splenocytes from AMP-CpG immunized mice exhibited a dramatic expansion of EBVpoly-specific cytokine^+^ CD8^+^ T cells in B*35:01 mice compared to soluble CpG comparators, with 65% and 42% cytokine^+^ cells, respectively (Supplementary Fig. S6). These CD8^+^ T cells exhibited strong polyfunctional effector phenotype with most cells secreting two or three cytokines (Supplementary Fig. S6). High frequencies of cytokine^+^ CD8^+^ T cell responses were also observed in expanded splenocytes from AMP-CpG immunized A*02:01 HLA transgenic mice as compared to soluble CpG immunized mice (Supplementary Fig. S6). Similar trends were observed for CD4^+^ T cells expanded and re-stimulated with gp350 OLPs, with an average of 17% and 11% cytokine^+^ CD4^+^ T cells in AMP-CpG and soluble CpG vaccinated mice, respectively (Supplementary Fig. S6). Cytokine^+^ CD4^+^ T cells were also increased after AMP-CpG immunization over adjuvant only controls for A*02:01 and B*08:01 HLA transgenic mice (Supplementary Fig. S6). Taken together, these data confirm the potent cellular immunogenicity of EBVpoly and further demonstrate the importance of effective adjuvant lymph-node targeting to optimally induce polyfunctional CD8^+^ and CD4^+^ T cells in mice.

### Humoral immune response

**Fig. 5:**
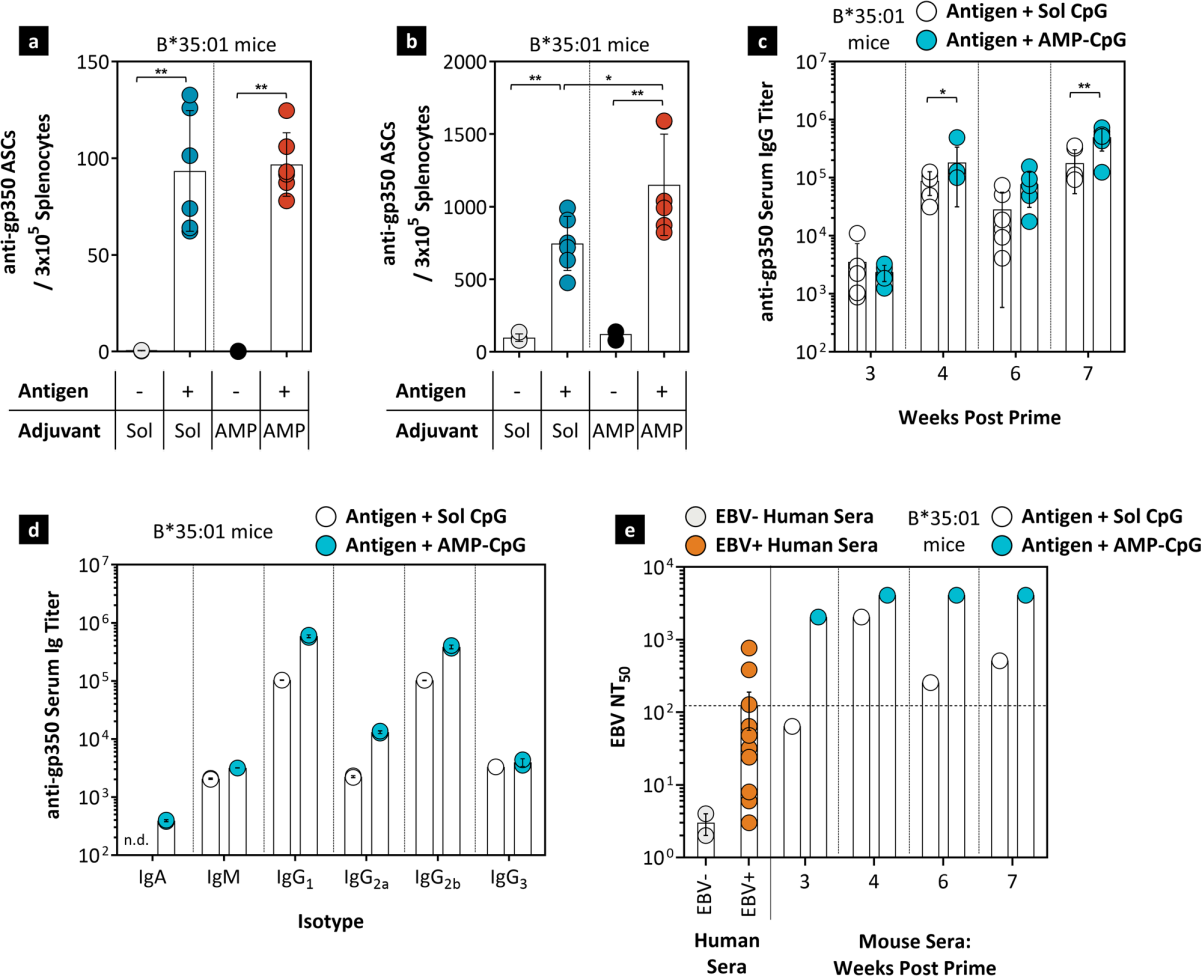
AMP-CpG induces strong serum IgG and neutralizing antibody responses targeting gp350. HLA-B*35:01 transgenic mice (*n* = 6 per vaccine group and *n* = 4 per control group) were immunized at weeks 0, 3, and 6 with 40 μg EBVpoly and 10 μg gp350 proteins admixed with 1.2 nmol soluble or AMP-CpG. Control groups were immunized with soluble CpG or AMP-CpG alone. Humoral responses to gp350 were assessed in splenocytes and serum from immunized mice at week 7 by ASC ELISPOT, ELISA or neutralization assay. Shown are **a** ex vivo ASC ELISPOT measured frequency of gp350-specific ASCs per 3 × 10^5^ splenocytes, **b** expanded ASC ELISPOT measured frequency of gp350-specific ASCs per 3 × 10^5^ splenocytes, **c** longitudinal serum IgG titers, **d** Ig subtype titers at week 7 in pooled serum samples, and **e** EBV-neutralizing antibody titers in EBV-seropositive and EBV-seronegative donors alongside longitudinal EBV neutralization titers in pooled serum samples (*n* = 6, serum samples pooled per vaccine group). Values depicted are mean ± standard deviation. Not detected (n.d.) values are shown on the baseline. Dotted line indicates the detected average level of EBV NT_50_ among EBV seropositive subjects. Data are representative of one experiment. *p < 0.05; **p < 0.01 by two-sided Mann-Whitney test. If statistics are not indicated, then the comparison was not significant. The exact *p*-values are as follows. **a** Soluble vaccine to soluble CpG-0.0095 and AMP vaccine to AMP-CpG-0.0095. **b** Soluble vaccine to soluble CpG-0.0095, AMP vaccine to AMP CpG-0.0095 and AMP vaccine to soluble vaccine-0.030. **c** AMP vaccine to soluble vaccine at 4 weeks-0.0260, AMP vaccine to soluble vaccine at 7 weeks-0.0087. Source data are provided as a Source Data file.

Potent IgG responses including neutralizing activity are critical to providing effective protection against primary EBV infection of B cells^46^. To evaluate gp350-specific B cell responses in B*35:01 transgenic mice, splenocytes were tested in a gp350-specific B cell ELISpot. Mice immunized with AMP-CpG or soluble CpG induced comparable numbers of gp350-specific antibody-secreting B cells when directly assessed ex vivo (Fig. 5a). Corresponding memory B cell responses were subsequently assessed by first inducing B cells to differentiate into antibody-secreting cells (ASCs) for 3 days before assaying for gp350-specific antibody-secreting cells by B cell ELISpot assay. Increased numbers of memory gp350-specific ASCs were induced after immunization with AMP-CpG as compared to soluble CpG (Fig. 5b) indicating enhanced memory response quality associated with AMP-CpG adjuvant administration. Three weeks after the initial immunization, both cohorts showed robust gp350-specific serum IgG responses. However, post-dose peak antibody responses at weeks 4 and 7 were significantly elevated among the AMP-CpG immunized cohort compared to the soluble CpG comparators (Fig. 5c). These trends were similar when assessed in A*02:01 and A*24:02 mice but no significant differences were observed in B*08:01 expressing animals (Supplementary Fig. S7). We also evaluated the induced Ig subclasses and found that B*35:01 transgenic mice immunized with AMP-CpG had similar IgM and IgG3 (Th1 Ig isotype) titers compared to mice immunized with soluble CpG, while IgA, IgG1 (Th2 Ig isotype)^47^ and IgG2a and IgG2b (Th1 Ig isotypes) titers were elevated in AMP-CpG immunized groups (Fig. 5d). Assessment in A*02:01, A*24:02, and B*08:01 expressing mice showed similar patterns of isotype induction with enhanced or equivalent titers observed after AMP-CpG immunization relative to soluble CpG comparators (Supplementary Fig. S7). Finally, neutralizing antibody activity was assessed through an EBV-induced human B cell proliferation assay. Sera from AMP-CpG immunized mice exhibited approximately 100-fold increased neutralizing titers compared to soluble CpG vaccinated mice three weeks after the prime dose (week 3) and 1 week post the second booster dose (week 7, Fig. 5e). By comparison, the observed activity in week 7 sera from AMP-CpG immunized mice exhibited 31-fold higher neutralizing antibody titers relative to those assessed in serum samples from EBV-seropositive individuals (Fig. 5e). Similar to the increases in cellular immune responses stimulated by AMP-CpG, these data further demonstrate the potential for enhanced adjuvant lymphatic targeting to promote potent humoral immunity including substantially increased neutralizing activity important for preventing nascent viral infection.

### Long-term maintenance of cellular and humoral immune responses

**Fig. 6:**
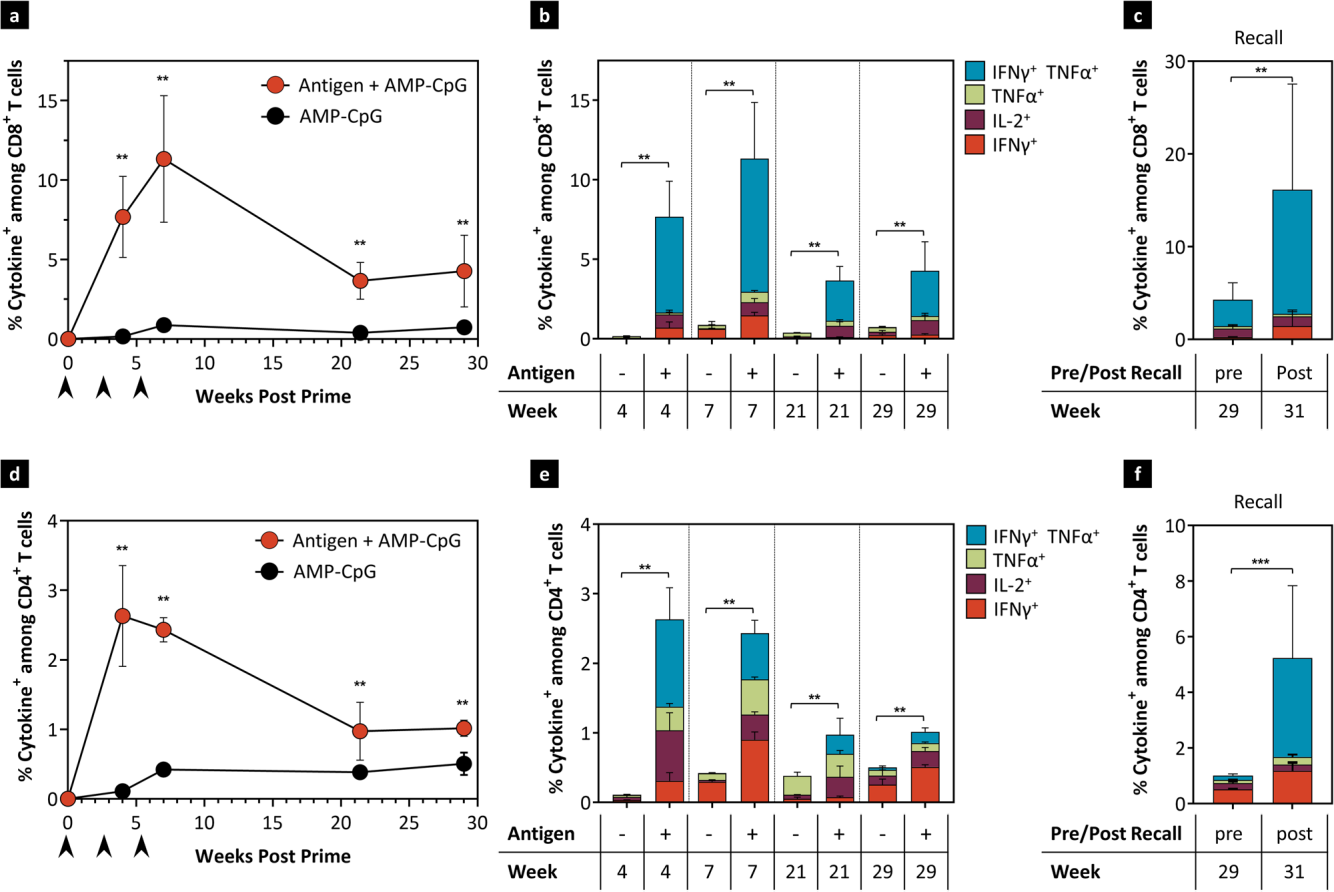
Vaccination with AMP-CpG maintains EBV-specific CD8^+^ and CD4^+^ T cells responses for >7 months. **a**–**f** HLA-B*35:01 transgenic mice (*n* = 9 per vaccine group and *n* = 5 per control group) were immunized at weeks 0, 3, and 6 with 40 μg EBVpoly and 10 μg gp350 proteins admixed with 1.2 nmol AMP-CpG and T cell responses were analyzed at long-term timepoints. The control group was immunized with AMP-CpG alone. Splenocytes were stimulated with **a**–**c** EBV CD8^+^ T cell peptides or **e**, **f** gp350 OLPs in an ICS assay. Shown are frequencies of total IFNγ, IL-2, and TNFα **a** CD8^+^ or **e** CD4^+^ T cells over time; polyfunctional cytokine^+^ **b** CD8^+^ or **f** CD4^+^ T cell frequencies at different timepoints; and post-boost week 31 **c** CD8^+^ or **f** CD4^+^ T cell response to a recall vaccination at week 30, compared to week 29 pre-boost responses. Data are representative of one experiment. Values depicted are mean ± standard deviation. Black arrows indicate immunization days. *p < 0.05; **p < 0.01; and ***p < 0.001 by two-sided Mann–Whitney test applied to cytokine^+^ T cell frequencies. If statistics are not indicated, then the comparison was not significant. The exact *p*-values are as follows. **a** AMP vaccine to AMP-CpG at week 4, 7, 21, and 29-0.0095. **b** AMP vaccine to AMP-CpG at weeks 4, 7, 21, and 29-0.0095. **c** Post recall to pre-recall-0.0016. **d** AMP vaccine to AMP-CpG at weeks 4, 7, 21, and 29-0.0095. **e** AMP vaccine to AMP-CpG at weeks 4, 7, 21, and 29-0.0095. **f** Post recall to pre-recall-0.0004. Source data are provided as a Source Data file.

Given the need for durable cellular immune responses against EBV to control the spread of latently infected B cells, circulating T cell responses were assessed longitudinally after three doses of EBV vaccine admixed with AMP-CpG or AMP-CpG adjuvant alone in HLA-B*35:01 transgenic mice. At all post-immunization time points, the EBVpoly-specific CD8^+^ T cell response in peripheral blood was significantly elevated in EBV-vaccinated mice compared to control mice. The peak of the EBVpoly-specific CD8^+^ cytokine^+^ T cell response was at week 7, one week after the second boost with subsequent contraction of the response through week 29. Importantly, responses were maintained for greater than 6 months post immunization at higher levels than the control group (Fig. 6a, b). The induced EBV-specific CD8^+^ T cells remained highly polyfunctional at week 29, with 72% of cells secreting 2 or 3 cytokines (Supplementary Fig. S8). In vitro stimulation of splenocytes to assess expansion of memory responses resulted in greatly increased frequency of EBVpoly-specific CD8^+^ T cells at the long-term timepoints (Supplementary Fig. S9). Mice from each cohort were recalled with a subsequent immunization at week 30 and the recall response was evaluated 1 week later, at week 31. Recall immunization increased the frequency of cytokine^+^ CD8^+^ T cells >4-fold over week 29 from 4% to 16% ex vivo, levels similar to earlier peak responses (Fig. 6c) with similar trends observed after expansion in vitro (Supplementary Fig. S10). Similar results were seen with gp350-specific CD4^+^ T cells, although the peak of the response was earlier than for CD8^+^ T cells, at week 4 (Fig. 6d, e). These CD4^+^ T cell responses were maintained through week 29 above adjuvant-only controls. Consistent with memory responses for CD8^+^ T cells, greatly increased frequencies of cytokine^+^ CD4^+^ T cells were observed following in vitro expansion with gp350 OLPs (Supplementary Fig. S9). The recall immunization also boosted gp350-specific CD4^+^ T cells five-fold over the pre-recall timepoint, from 1% to 5% cytokine^+^ CD4^+^ T cells ex vivo (Fig. 6f) while expanded responses were comparable to earlier peak levels (Supplementary Fig. S10). When compared to mice immunized with soluble CpG, CD8^+^ T cell responses were 2-fold higher for AMP-CpG immunized mice, whereas CD4^+^ T cell responses were similar between the two cohorts (Supplementary Fig. S11). These data indicate that immunization with AMP-CpG stimulates long-lived CD4^+^ and CD8^+^ T cell responses including robust memory T cells capable of rapid expansion and polyfunctional cytokine secretion in response to subsequent exposure to EBV immunogens.

**Fig. 7:**
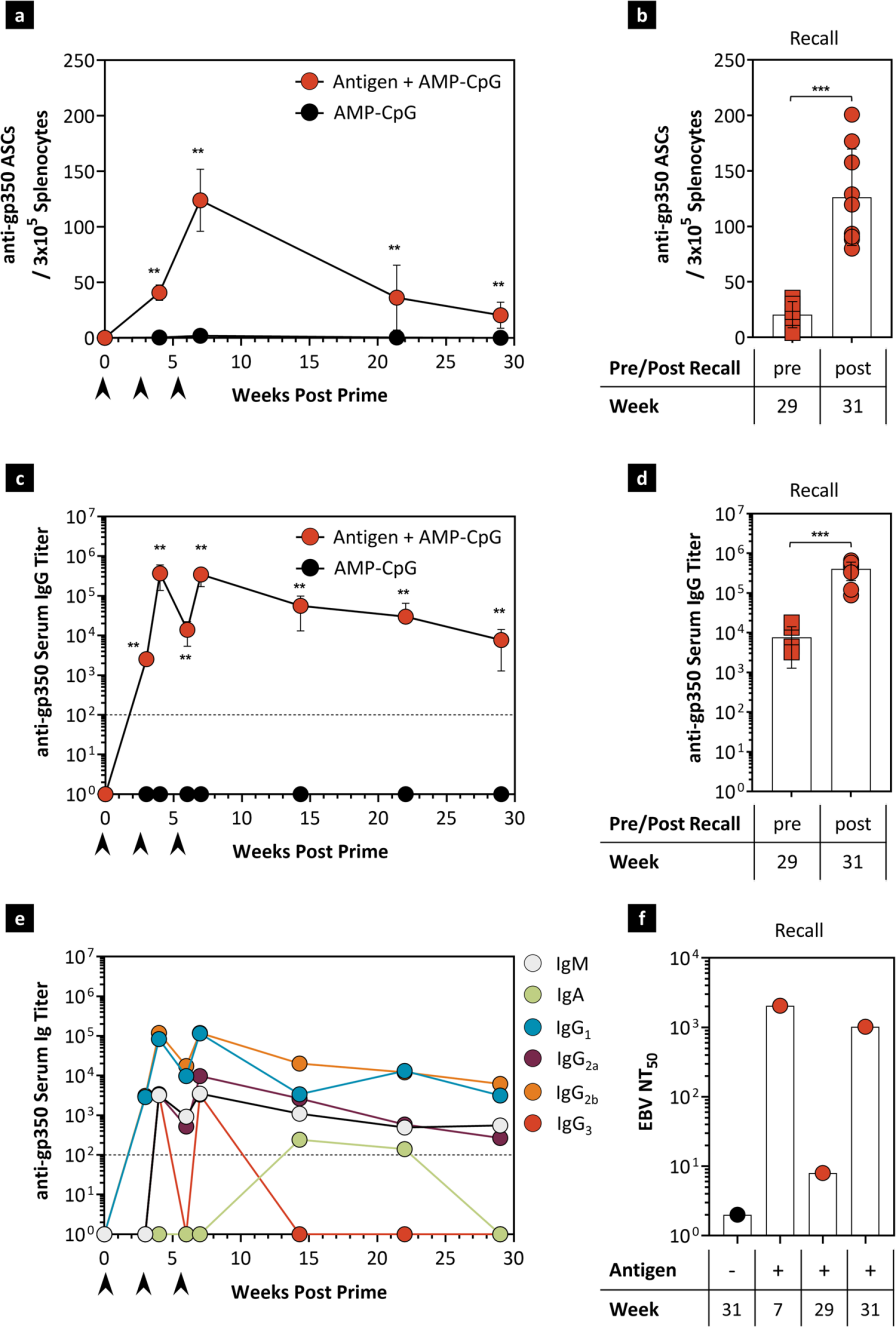
Vaccination with AMP-CpG induces durable gp350-specific IgG responses. HLA-B*35:01 transgenic mice (*n* = 9 per vaccine group and *n* = 5 per control group) were immunized at weeks 0, 3, and 6 with 40 μg EBVpoly and 10 μg gp350 proteins admixed with 1.2 nmol AMP-CpG. The control group was immunized with AMP-CpG alone. Humoral responses to gp350 were assessed in splenocytes and serum from immunized mice at week 7 by ASC ELISPOT or ELISA assay. Shown are **a** ex vivo ASC ELISPOT measured frequency of gp350-specific ASCs per 3 × 10^5^ splenocytes, **b** week 31 ex vivo ASC recall response to a recall vaccination at week 30, **c** longitudinal serum IgG titers, **d** week 31 IgG titers to a recall vaccination at week 30, **e** longitudinal Ig subtype titers in pooled serum samples, and **f** week 31 recall EBV neutralization titers to a recall vaccination at week 30, compared to 29 pre-boost responses using pooled serum samples. Data are representative of one experiment. Values depicted are mean ± standard deviation. Black arrows indicate immunization days. Dotted lines in **c, e** indicate the assay LOD. *p < 0.05; **p < 0.01; ***p < 0.001 by two-sided Mann–Whitney test. The exact p-values are as follows. **a** AMP vaccine to AMP-CpG at week 4, 7, 21, and 29-0.0095. **b** Post recall to pre-recall-0.0004. **c** AMP vaccine to AMP-CpG at week 3, 4, 6, 7, 14, 21, 29-0.0095. **d** Post recall to pre-recall-0.0004. Source data are provided as a Source Data file.

Durable antibody responses are also important to prevent infection and control the expansion of latently infected B cells. We evaluated gp350-specific B cell and anti-gp350 antibody responses at long-term timepoints. Splenic gp350-specific ASCs were maintained at significantly elevated levels through week 29, 7 months after the initial immunization with AMP-CpG (Fig. 7a). A recall immunization at week 30 significantly increased ASC responses above week 29 pre-boost levels, to similar responses as to the peak at week 7 (Fig. 7b). Memory ASCs were also durably elevated through 6 months and a subsequent recall at week 30 boosted above week 29 pre-boost levels (Supplementary Fig. S9). Anti-gp350 IgG titers were maintained well above control group levels through week 29 while the recall response increased IgG titers to similar peak levels observed at week 4 (Fig. 7c, d). Evaluation of Ig subtypes showed that IgM, IgG1, IgG2a and IgG2b were durable for 7 months, but IgG3 titers were transiently elevated before decline by week 14 (Fig. 7e). EBV neutralization activity of serum collected throughout the study showed a peak at week 7 consistent with the observed peak in ASC response (Fig. 7f). Assessment at week 29 showed maintenance of neutralizing activity above adjuvant-only background levels, and these were increased to near-peak levels at week 31, 7 days following recall. Thus, immunization with AMP-CpG rapidly generated robust neutralizing antibody responses which are maintained for at least 7 months and are quickly boosted after recall exposure to EBV immunogens.

### In vivo protection against EBV-induced B cell lymphoma following adoptive immunotherapy with EBVpoly-stimulated T cells with or without serum antibodies from vaccinated mice

**Fig. 8:**
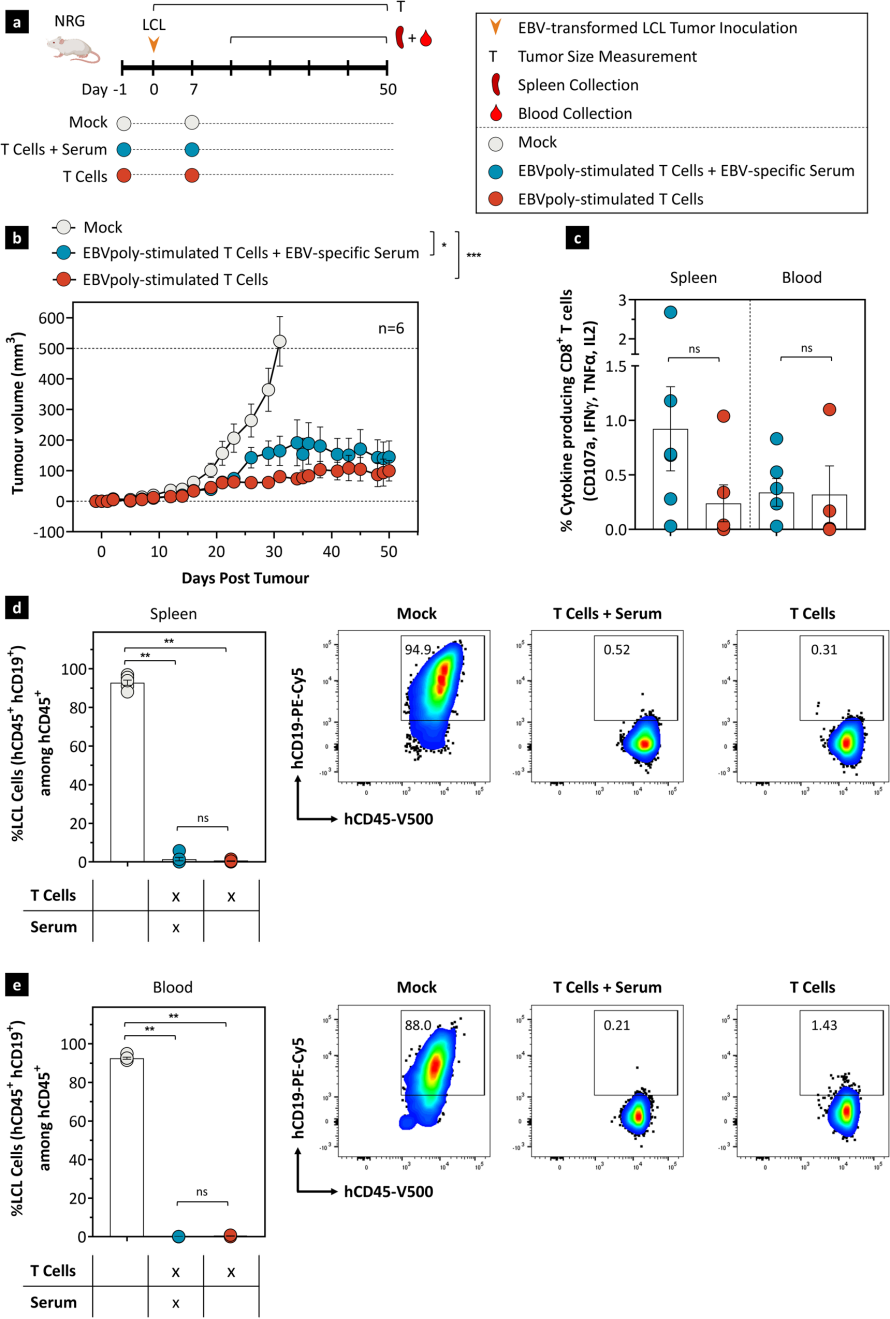
Adoptive immunotherapy with EBVpoly-stimulated T cells with or without serum antibodies from vaccinated mice confers protection against EBV-associated B cell lymphoma in vivo. **a** Study schema. Three groups of 6–8 week old NRG mice (*n* = 6/group) were treated with PBS (100 µL i.v.), EBVpoly-stimulated T cells (2 × 10^7^ cells/mouse i.v.) in combination with serum from vaccinated mice containing gp350-specific antibodies (100 µL/mouse i.p.) or EBVpoly-stimulated T cells alone (2 × 10^7^/mouse i.v.) on day −1. On day 0, these mice were engrafted with EBV-transformed lymphoblastoid cells (LCLs) (4 × 10^6^ cells/mouse s.c.). Seven days after LCL engraftment, mice were treated again as outlined for day −1 and monitored for the outgrowth of EBV lymphomas. **b** shows tumor volume measured using Vernier callipers. Each data point shows mean ± SEM of tumor volume in mice treated with PBS (Group 1), EBVpoly-stimulated T cells in combination with serum antibodies (Group 2) or EBVpoly-stimulated T cells alone (Group 3). Dotted line indicates maximum ethical limit of tumor volume (500 mm^3^). **c** shows frequencies of EBV-specific CD8^+^ T cells in splenocytes and in blood of animals from groups 2 and 3 on day of killing. **d**, **e** frequencies of human B cells in spleen and blood at the completion of follow up and animal killing. Data are representative of one experiment with *n* = 6 per group. Values depicted are mean ± s.e.m. not significant (ns), *p < 0.05, ***p < 0.001, and ****p < 0.0001 by two-sided Mann–Whitney test. The exact p-values are as follows. **d** PBS to T cells + Serum-0.0022 and PBS to T cells-0.0022. **e** PBS to T cells + Serum-0.0022 and PBS to T cells-0.0095. Source data are provided as a Source Data file. Created with BioRender.com.

To assess in vivo efficacy of T cell and antibody responses representative of those induced by EBV vaccination, we evaluated an EBV-induced lymphoma model in NOD.Rag1KO.IL2RγcKO (NRG) mice. These animals were treated with (1) PBS (Mock), (2) EBVpoly-stimulated T cells in combination with serum from vaccinated mice containing gp350-specific antibodies or (3) EBVpoly-stimulated T cells alone on day −1 and then engrafted subcutaneously with EBV-transformed lymphoblastoid cells (LCLs). Seven days after LCL engraftment, mice were treated again as outlined for day −1 and monitored for the outgrowth of EBV lymphomas (Fig. 8a). While untreated NRG mice developed rapidly progressing tumors requiring euthanasia for most animals by day 30, mice treated with either EBVpoly-stimulated T cells in combination with gp350-specific antibodies or EBVpoly-stimulated T cells alone significantly inhibited outgrowth of EBV-induced lymphomas (Fig. 8b). Interestingly, some of these animals also showed persistence of adoptively transferred EBVpoly-stimulated CD8^+^ T cells in spleen and blood with polyfunctional production of multiple cytokines (IFNγ, TNFα, IL-2) and expression of degranulation marker CD107a (Fig. 8c). Consistent with progressive subcutaneous lymphoma growth, Mock-treated animals developed significant populations of human B cells (consistent with EBV-transformed LCLs) in spleen and peripheral blood. In contrast, animals treated with EBVpoly-stimulated T cells with or without gp350-specific antibodies showed very low or undetectable numbers of human B cells in spleen and peripheral blood (Fig. 8d and e). Collectively, these data indicate that development of durable EBV-specific CD8^+^ T cell responses stimulated with EBVpoly with or without gp350-specific antibodies are able to effectively control the spread of EBV-associated lymphoma in vivo.

## Discussion

EBV is classified as a class I carcinogen due to its high oncogenic potential and association with several lymphoid and epithelial cancers^48^. More recently, a definitive link has been established between EBV infection and subsequent MS^3,49^. Hence, prophylactic and therapeutic vaccines against EBV would have a substantial public health and economic impact. In addition, the success of prophylactic vaccines against hepatitis B and oncogenic strains of human papillomavirus in prevention of cancers has triggered interest in the possibility of primary prevention of EBV-associated cancers through vaccination. However, despite considerable efforts no EBV vaccine has been licensed for human use^50,51^.

Previous studies in healthy virus carriers and patients with EBV-associated diseases have provided critical insights on the natural immune response to EBV. In the infected host, EBV establishes a complex life cycle characterized by differentially expressed proteins. Expression of viral proteins in specific stages of the viral cycle and their associated molecular interactions with host cells plays an important role in the establishment of latent EBV-infection in B cells. Primary and latent EBV infection is controlled through a broad array of immune effector pathways which includes neutralizing antibodies, natural killer cells, CD8^+^ cytotoxic T cells and CD4^+^ helper T cells directed against multiple EBV antigens^52,53^. Individuals with defective humoral and/or cellular immunity can develop life-threatening complications leading to uncontrolled proliferation of EBV-infected cells. This is best exemplified by outgrowth of EBV-infected B cells in immunocompromised transplant recipients which can be reversed by adoptive transfer of EBV-specific T cells^54^. These observations and accumulating evidence from adoptive immunotherapy studies suggest that an ideal vaccine should prevent acute symptoms of primary EBV infection through the induction of robust humoral and cellular immunity including neutralizing antibodies and EBV-specific CD4^+^ and CD8^+^ T cell responses. Moreover, in patients where primary infection does occur, induction of persistent immune responses, including long-lived T cells, may prevent development of EBV-associated malignancies and autoimmune diseases which emerge long after the primary infection.

To achieve this goal, we have designed a multi-antigen-specific protein subunit vaccine which includes EBV-encoded gp350 and EBVpoly recombinant proteins. EBV gp350 is a predominant protein element of the EBV viral capsid responsible for mediating viral entry to host cells through interaction with complement receptor 2 (CR2/CD21). Prior clinical studies have established the potential for vaccine-induced gp350-targeted neutralizing antibody responses to inhibit viral infection resulting in reduction of acute IM^16^. However, previous response levels were not effective at preventing EBV infection, suggesting that more potent neutralizing activity or concomitant action through vaccine-induced cellular immunity may be necessary to improve prophylactic efficacy^55^. Accordingly, EBVpoly is an engineered recombinant protein, precisely designed to encode 20 different conserved immunodominant CD8^+^ T cell epitopes derived from multiple EBV lytic and latent antigens. These epitopes are restricted through 16 different HLA class I alleles which covers >92% of the world-wide population with an average recognition of 2.66 epitopes predicted for immunized individuals based on HLA haplotype analysis^42^. Inclusion of highly conserved epitopes from both lytic and latent antigens which are restricted to common HLA alleles is intended to allow broader coverage amongst different ethnic groups. In vitro stimulation of human PBMCs from healthy virus carriers with EBVpoly protein expanded polyfunctional CD8^+^ T cells which were directed to 2–5 epitopes derived from both latent and lytic EBV antigens (Fig. 2). These observations highlight that the T cell responses expanded by EBVpoly protein are dependent on the HLA alleles expressed by an individual. Furthermore, in vivo studies in HLA transgenic mice also demonstrated that in addition to T cell responses directed to EBVpoly protein, strong CD4^+^ and CD8^+^ T cell responses were also generated against gp350 suggesting a diverse response could be achieved in potential human vaccinees. Previous studies have shown EBV-specific T cells play a crucial role in controlling the outgrowth of EBV-infected cells^56^ and adoptive immunotherapy with these effector T cells can offer therapeutic benefit against EBV-associated malignancies and multiple sclerosis^57–60^. Taken together, our vaccine formulation based on a combination of both EBVpoly and gp350 is designed to induce broad multi-antigen-directed T cell immunity directed to both latent and lytic antigens.

We evaluated the immunogenicity of gp350 and EBVpoly proteins in combination with a lymph node-targeted molecular adjuvant, AMP-CpG, a lipid-modified TLR-9 agonistic DNA oligonucleotide, which is efficiently delivered to professional APCs in the lymph nodes and induces robust immune responses against co-administered protein immunogens^38,61^. Previous studies have shown that the AMP lipid domain can mediate non-covalent binding of AMP-CpG to endogenous albumin in peripheral tissue to enable albumin to serve as an efficient chaperone promoting improved biodistribution into draining lymph nodes^62^. Indeed, lymph node proteomic analysis showed that AMP-CpG administration induced robust activation of local innate immune responses. Notably, these were coincident with the timing of immunogen entry to lymph nodes suggesting the potential for AMP-adjuvant to promote concerted exposure of lymph node immune cells to activation stimulus with antigen. Soluble CpG adjuvant however failed to optimally promote lymph node antigen accumulation or development of a robust inflammatory milieu. These trends were most notable in relation to EBVpoly, which was minimally present in draining lymph nodes when administered with soluble CpG. These changes in antigen biodistribution are consistent with past studies observing adjuvant-driven mechanisms for improving lymph node accumulation of co-administered protein antigens; for example (i) local recruitment, antigen acquisition, and lymphatic transit of phagocytes, (ii) enhancement of afferent lymph flow, and (iii) optimization of antigen capture by lymph node resident innate immune cells have been previously reported^45,63,64^. These mechanisms may be promoted by AMP-CpG given the significant improvement in EBVpoly accumulation to draining lymph nodes associated with AMP-CpG relative to soluble CpG comparators. The lack of similar improvements with gp350 suggests the underlying mechanisms may be dependent on immunogen size or overall physical properties. Indeed, larger macromolecules are known to exhibit impaired convection through extracellular matrix, thus restricting access to the initial lymphatic vessels^65^, a prerequisite for improved lymph node uptake mediated by adjuvant-driven enhancements to passive immunogen lymphatic transport and lymph node retention.

Consistent with enhancements to lymph node antigen trafficking and concerted upregulation of inflammatory proteomic signatures, EBV vaccine formulated with AMP-CpG induced robust EBV-specific cellular and humoral immune responses in multiple HLA transgenic mice and these responses were significantly higher when compared to soluble CpG adjuvanted comparators. Cellular immune responses were polyfunctional with a majority of T cells expressing two or more cytokines and included both CD8^+^ and CD4^+^ T cells directed to latent and lytic antigens and to gp350. Notably, previous studies have shown that EBV EBNA1-specific CD4^+^ T cells play an important role in prevention of early-phase EBV-induced B cell proliferation^66^. Antibody responses induced by the AMP-CpG formulated EBV vaccine showed 16-25-fold higher neutralizing titers when compared to vaccine formulation with soluble CpG. Moreover, neutralizing antibody titers in AMP-CpG vaccinated mice were >30-fold higher than those measured in healthy EBV virus carriers. Previous studies have shown that acquisition of EBV-specific neutralizing antibodies is coincident with the recovery from acute IM^67,68^, and elevated titers of gp350 antibodies produced over time during infectious mononucleosis inversely correlated with the severity of the disease^69^. Antibody isotype may also contribute to the effectiveness of gp350-specific responses to EBV infection. Hybridomas producing gp350-specific IgG1 or IgG2a antibody isotypes demonstrated higher neutralizing activity against EBV infection in vitro^70^ and during acute IM, IgG1 antibodies mediate binding and functional antibody activity throughout infection; these isotypes are a major mediator of ADCC and neutralization^71,72^. Furthermore, serum gp350-specific IgA antibody titers in infants correlate with neutralization of EBV infection of B cells; likewise, reduced gp350-specific IgA antibody titers increased risk of coinfection with a second strain of EBV^73^. In this study antibody isotype analysis revealed AMP-CpG formulated EBV vaccine generated a mixture of IgA, IgM, IgG1, IgG2a, IgG2b, and IgG3 gp350-specific antibody isotypes, suggesting the potential for immunization with AMP-CpG to promote enhanced immune control of primary infection and prevention of progression to symptomatic IM.

Another important aspect of EBV vaccine development is the long-term persistence of immune responses which are not only crucial for durable prevention of primary infection but may also play an important role in blocking the development of EBV-associated malignancies and autoimmune disorders. Both cellular and humoral immune responses induced by AMP-CpG formulated EBV vaccine were sustained for over 7 months in mice. Interestingly, the anti-gp350 IgG titers largely remained unchanged during the follow-up period. week 29 analysis revealed a trend of decreased neutralizing activity; however, recall EBV immunization with AMP-CpG restored neutralizing antibody responses to peak levels observed at week 7 indicating the presence of durable memory humoral immune responses. While longitudinal assessments showed a decline in the ex vivo frequency of antigen-specific CD8^+^ and CD4^+^ T cells post vaccination, ex vivo re-stimulation with HLA-matched peptide epitopes rapidly expanded these T cells, with a majority showing a polyfunctional profile. This was consistent with the rapid expansion of T cell responses in vivo following recall immunization of vaccinated mice which restored response frequencies to peak levels. These results confirm the presence of durable functional memory responses following vaccination with AMP-CpG.

Long-term persistence of cellular and humoral immunity may provide more robust protection against EBV-associated diseases. Thus, we tested the protective efficacy of EBVpoly-stimulated CD8^+^ T cells and gp350-specific antibodies against EBV-associated B cell lymphoma in NRG mice. Adoptively transferred EBVpoly-stimulated T cells with or without gp350-specific antibodies similarly controlled the progression of EBV lymphoma and associated outgrowth of EBV-transformed LCLs in spleen and peripheral blood. Together these results confirm the activity of EBV-specific CD8^+^ T cells against EBV-transformed cancers. It is also important to emphasize that while vaccine formulations based on EBV glycoproteins (gp350, gH/gL, and gH/gL/gp42) have shown some protection against EBV infection in humanized mice^11^, it is unlikely that the antibodies directed to these glycoproteins alone can offer protection against latent EBV infection. In contrast, previous studies have clearly demonstrated that adoptive immunotherapy with latent antigen-specific T cells can reverse the outgrowth of EBV malignancies and offer clinical benefit to patients with progressive MS^60,74–76^. Therefore, vaccine-induction of cellular immunity alongside neutralizing antibody responses with broad specificity against targets spanning the viral life cycle offers an attractive opportunity to improve prevention of disease associated with primary infection (IM) and chronic latent infection (MS and EBV-driven malignancy).

Collectively, the data presented here clearly demonstrate that EBV protein subunit vaccine formulated with AMP-CpG can generate robust virus-specific cellular and humoral immunity which is persistent and capable of rapid expansion upon recall through exposure to EBV antigens. These studies provide an important platform for future clinical assessment of this vaccine formulation in human volunteers.

## Methods

### EBV polyepitope design, protein expression, and purification

Twenty different CD8^+^ T cell epitopes derived from lytic and latent EBV antigens were selected (Fig. 1b). The carboxyl terminus of each epitope was joined by a proteasome liberation amino acid sequence (AD or K or R). Proteasome liberation amino acid sequences improve the immunogenicity of CD8^+^ T cell epitopes by enhancing proteasomal processing of the polyepitope protein by APCs^77^. To achieve high level of EBVpoly protein expression, the amino acid sequence of the EBVpoly construct was translated into DNA sequence using optimized *E. coli* codons and the protein-encoding DNA sequence was synthetically constructed and cloned into an isopropyl–β-d-thiogalactopyraniside (IPTG) inducible plasmid, pJexpress 404 (Atum Bio, CA, USA). Chemically competent BL21-codonPlus (DE3) RP *E. coli* cells (Agilent Technologies, CA, USA) were transformed with the inducible EBVpoly expression plasmid. To initiate protein expression culture was scaled up to 3L (Terrific Broth containing ampicillin) and then EBVpoly protein expression was induced by adding 1 mM IPTG per mL of culture and incubating for 6 hours at 25 ^o^C. At the end of the induction phase, the culture was harvested, and cells were lysed. Due to the high hydrophobic nature of the linear CD8^+^ T cell epitopes, the induced EBVpoly protein was aggregated in the form of inclusion bodies (IBs). Inclusion bodies were further purified by washing them with TE buffer (25 mM Tris and 5 mM EDTA) and EBVpoly protein solubilized in 50 mM NaH_2_PO_4_, 10 mM Tris, 5 mM DTT, 8 M urea, pH 9.5 buffer. The pH of the solubilized protein was then decreased to pH 7.0. To eliminate the host DNA and lipid contaminants solubilized protein was passed through the Q Sepharose FF column (Cytiva Sweden AB, Uppsala Sweden) and then EBVpoly was purified using phenyl sepharose column (Cytiva Sweden AB, Uppsala Sweden).

### gp350 expression and purification

EBV gp350 nucleotide sequence encoding the extracellular domain was cloned in to a mammalian expression system and splice site mutations were carried out as outlined previously^78^. The gp350 encoding vector was then transfected into CHO cells (ATCC, cat#CCL61, not authenticated). Transfected CHO cell culture was scaled up and gp350 protein was purified from cell culture supernatant using cation, anion exchange, and size exclusion chromatography techniques in a sequential manner^79^. Final purified gp350 was stored in 20 mM histidine and 6% trehalose pH 6.0 buffer and purified protein was characterized using SDS-PAGE and Western blot analysis.

### Animals and study design

Ethics approval to conduct animal experiments were obtained from QIMR Berghofer Medical Research Institute Animal Ethics committee under project number P2241 or from Charles River Accelerator Development Lab Institutional Animal Care and Use Committee (IACUC) under Protocol# 2021-1259. All human HLA expressing mice (HLA A2, HLA A24, HLA B8 and HLA B35) were obtained from Institut Pasteur (Paris, France). The HLA transgenic mice were generated on C57BL/6 background by fusing HLA α1α2 H chain domains with a mouse α3 domain and covalently linked to human β2 macroglobulin^80^. Mice were bred and maintained under pathogen-free environment at the QIMR Berghofer animal facility. Two groups of 6–8 weeks old female mice (*n* = 6) for each HLA transgene were immunized with 3 doses of EBV vaccine comprising 40 µg of EBVpoly and 10 µg of gp350 proteins, formulated with either 1.2 nmol AMP-CpG-7909 (5′AMP-tcgtcgttttgtcgttttgtcgtt-3′; Elicio Therapeutics, Boston, MA, USA) or 1.2 nmol CpG-7909 (InvivoGen, San Diego, CA, USA). Another two groups of mice (*n* = 4) were injected with 3 doses of 1.2 nmol AMP-CpG-7909 or 1.2 nmol CpG-7909 to serve as placebo (adjuvant-alone control) group. All injections were administered subcutaneously, 50 µl at each side of the tail base (100 µl total) on day 0, weeks 3 and 6. The mice were tail bled at weeks 3, 4, and 6, and were finally killed at week 7. For long-term immunogenicity evaluation, multiple groups of HLA B35 mice were immunized with vaccine (*n* = 36) or control (*n* = 24) formulations as mentioned above. Mice were killed at weeks 4, 7, 21 and 29. To determine the recall response, mice (*n* = 9) were immunized with a booster dose at week 30 and these mice were killed after 7 days, at week 31. Blood, spleen, inguinal and axillary lymph nodes were collected to assess EBV-specific humoral and cell-mediated responses using ICS assays, gp350 ELISpot, ELISA, and neutralizing antibody assays.

### Biodistribution and lymph node activation assays

AlexaFluor (AF) 594-maleimide fluorophore (Thermo Fisher Scientific, Cat No. A10256) was thioether-coupled to EBVpoly protein at pH 7.0-7.5 in the presence of reducing agent TCEP [tris(2-carboxyethyl)phosphine]. The reaction was allowed to proceed at RT for 2 hours and protein precipitates were formed. After centrifugation, the protein pellet was resuspended in 6 M guanidine hydrochloride solution at 2 mg/ml. In contrast, cysteine residues of EBV gp350 protein are not accessible even after TCEP treatment. Therefore, thiol groups were introduced onto the gp350 protein. SPDP-Peg4-NHS (Quanta Biodesign, Cat No. 10374) was added to the gp350 PBS solution and incubated at RT for 30 min. gp350-SPDP was treated with TCEP and AF647-maleimide (Thermo Fisher Scientific, Cat No. A20347) for 2 h at RT. gp350-AF647 was purified on PD-10 columns and subsequently concentrated on a lyophilizer and resuspended in PBS at 2 mg/ml. 6–8 week old, female C57Bl/6 J mice (*n* = 6 per group; Jackson Laboratory, ME, USA) were immunized with 8 µg EBVpoly-AF594 and 10 µg gp350-AF647 admixed with 1.2 nmol soluble or AMP-CpG. Fluorescent negative controls received equivalent amounts of AMP-CpG and unlabeled antigen. Mock-treated animals were administered vehicle alone (25 mM glycine, pH 4.5). Lymph nodes were harvested from immunized animals 24 and 48 h post vaccine administration and imaged ex vivo using the In Vivo Imaging System (IVIS) Spectrum CT. AF594 fluorophore was excited at 570 nm and detected at 620 nm, and AF647 fluorophore was excited at 640 nm and detected at 680 nm. Lymph nodes were further processed for proteomic analysis by Luminex to determine their cytokine/chemokine content. Protein Extraction Buffer (Invitrogen, cat# EPX-9999-000) contained Mini protease inhibitor cocktail (Roche, cat # 53945000) and HALT phosphatase inhibitors (diluted 1:100, Thermo Fisher Sci cat# 78442). Lymph nodes were homogenized using a TissueLyser II (Qiagen). Cleared lysates were analyzed with Luminex Cytokine and Chemokine kits (EMDMillipore, cat# MCYTOMAG-70K and #MECY2MAG-73K) according to the manufacturer’s instructions. Analyte concentrations (pg/mL) in treatment groups were analyzed for significance compared to mock by ANOVA, followed by Dunnett test for multiple comparisons. False Discovery Rate analysis was applied using a 2-stage, step-up Benjamini, Krieger, and Yukatiele analysis conducted with *Q* = 5%.

### Evaluation of EBVpoly immunogenicity in human PBMCs

The study was approved by QIMR Berghofer Medical Research Institute Human Research Ethics Committee under project number P2282 and all healthy volunteers who offered blood samples gave written informed consent. PBMC from six different HLA-mapped, EBV-seropositive, healthy donors were stimulated with 25 µg of EBVpoly protein for 1 h at 37 °C. Then, cells were washed with RPMI supplemented with 10% FCS and cultured for 14 days to allow for T cell expansion; cultures were supplemented with medium containing RPMI and human recombinant IL-2 on days 2, 5, 8, and 11.

Following in vitro expansion of EBV-specific CD8^+^ T cells from healthy seropositive donors, cells were washed and then stimulated with 0.2 µg/mL of HLA matching peptides in the presence of human CD107a antibody conjugated to FITC (BD Pharmingen cat# 555800, 1:10), Golgiplug™ and Golgistop™ (BD Biosciences; CA, USA) for 5 h at 37 °C and 6.5% CO_2_. Cells were washed twice, then incubated with Live/Dead™ near IR (Invitrogen, cat# L34976, 1:250), Pacific Blue™-conjugated anti-CD4 (BD Pharmingen, cat# 558116, 1:200) and PerCP-Cy5.5-conjugated anti-CD8 (eBioscience, cat# 45008842,1:400). Cells were fixed and permeabilized using a BD Cytofix/Cytoperm™ kit (BD Biosciences; CA, USA). Then cells were incubated with PE-conjugated anti IL-2 (eBioscience, cat# 12702942, 1:50), APC-conjugated anti TNFα (BioLegend, cat# 502912, 1:200), and AF700-conjugated anti IFNγ (BD Biosciences cat# 557995, 1:50) to determine intracellular cytokine secretion. Cells were acquired on a BD FACSCanto™ II and data was analyzed (Supplementary Fig. S12) using FlowJo™ software (Becton, Dickinson and Company, OR, USA).

### gp350 ELISA

Serum total anti-gp350 antibody was evaluated by an enzyme-linked immunosorbent assay (ELISA). Briefly, immunosorbent 96-well plates were coated with 50 µL of recombinant EBV gp350 protein (2.5 µg/mL of gp350 protein diluted in phosphate buffer saline) and plates were incubated at 4 °C overnight. Plates were washed with phosphate buffer saline containing 0.05% Tween 20 (PBST) and then blocked with 5% skim milk. Serially diluted serum samples were added and incubated for 2 h at room temperature. After washing with PBST, plates were incubated with HRP-conjugated sheep anti-mouse Ig antibody (to determine total antibody response) (SouthernBiotech, cat# 1010-05, 1:1000) for 1 hour. These plates were washed and incubated with 3,3’,5,5’-Tetramethylbenzidine (TMB) substrate solution (Invitrogen, cat#00420156) for 10 min and then color development was stopped by adding 1 N HCl. Optical density (OD) at 450 nm was measured using an ELISA Biotek Power Wave Plate reader. The OD_450_ nm value of 0.5 was considered as baseline value and above 0.5 was regarded as positive response. The maximum dilution to give a positive result was used as the endpoint antibody titer.

### Antibody isotype analysis

Briefly, immunosorbent 96-well plates coated with recombinant gp350 were processed as described above and incubated with HRP-conjugated goat anti-mouse IgA (SouthernBiotech, cat# 1010-05, 1:1000), IgM (SouthernBiotech, cat# 1020-05, 1:1000), IgG1 (SouthernBiotech, cat# 1070-05, 1:1000), IgG2a (SouthernBiotech, cat# 1080-05, 1:1000), IgG2b (SouthernBiotech, cat# 1090-05, 1:1000), or IgG3 (SouthernBiotech, cat# 1100-05, 1:1000) antibody for 1 h. Plates were subsequently washed and incubated with TMB substrate solution for 10 minutes followed by 1 N HCl and analysis using an ELISA Biotek Power Plate reader and endpoint antibody titer was calculated as mentioned above.

### EBV neutralization assay

Equal volumes of mouse sera from independent animals were pooled for each group and timepoint to assess the ability to neutralize EBV using an EBV-induced B cell proliferation assay. In parallel assays, human serum samples from EBV-seropositive or EBV-seronegative healthy donors were assessed to compare neutralizing antibody titers with those observed in immunized mice. Briefly, the pooled mouse serum samples or human serum samples were heat inactivated at 56 ^o^C for 30 minutes. The samples then were serially diluted in duplicates, in 2-fold dilutions (from 1:2 to 1:4096 dilution), in 25 µL volumes in a 96-well U-bottom well plate. The B95-8 EBV viral isolate was added to the diluted serum samples in a 25 µL volume (50 µL/well total). The serum/virus mixture was incubated for two hours at 37 °C. PBMCs (100,000 cells in 50 µL/well) from EBV-seronegative donors labeled with CellTrace^TM^ Violet (Invitrogen, cat# C34557, 1:1000) were added and then incubated for one hour at 37 °C and 6.5% CO_2_. Cells were washed and incubated for 5 days at 37 °C and 6.5% CO_2_ to allow infection and proliferation of B cells from EBV seronegative donors. On day 5, cells were stained with Live/Dead™ near IR (Invitrogen, cat# L34976, 1:250), APC anti-human CD3 (BD Biosciences, cat# 340440, 1:25), PE-Cy5 anti-human CD19 (BD Pharmingen, cat# 555414, 1:25). Cells were acquired on a BD FACSCanto™ II and data was analyzed using FlowJo™ software. The maximum dilution to reduce the number of proliferating B cells by more than 50% compared to the control (i.e., PBMC infected with B95-8 EBV with no serum) was used as neutralization titer.

### B cell ELISpot

B cell ELISpot analysis was carried out using mouse IgG ELISpot kit (MAbtech AB, cat# 3825-2 A). PVDF ELISpot plates were treated with 70% ethanol, washed five times with distilled water, coated with 100 µL/well EBV gp350 protein (25 µg/mL) or anti-IgG antibody (15 µg/mL) as a positive control and incubated overnight at 4 °C. Plates were blocked with DMEM containing 10% serum and 3 × 10^5^ cells/well, in triplicate from each mouse, was added and then incubated for 18 hours in a 37 °C humidified incubator with 5% CO_2_. Cells were removed and plates were washed. Detection anti-IgG antibody conjugated to HRP (1 µg/mL) was added to each well and incubated for 2 hours at room temperature and then washed. Streptavidin-ALP (1:1000) was added to each well and incubated at room temperature for 1 h, followed by washing and treating plates with substrate solution containing BCIP®/NBT (Sigma-Aldrich, cat# SLCL7183, 100 µL/well) until color development was prominent. Color development was stopped by washing plates with water and plates were kept for drying overnight before counting spots in an AID ELISpot reader.

To measure memory B cell response, splenocytes (5 ×10^5^) from vaccine and control mice were activated with a mixture comprising the TLR7/8-agonist, R848 (resiquimod), and recombinant mouse IL-2 for three days in 24-well plate. Cells were washed three times and then counted. 2.5 × 10^4^ cells were transferred to respective wells in triplicates. The ELISpot was carried out as described above. The number of spots was counted in an AID ELISpot reader and the number of positive spots was normalized to calculate ASCs per 3 × 10^5^ splenocytes.

### Ex vivo intracellular cytokine staining assay

Splenocytes were stimulated with either 0.2 μg/mL of HLA B35 (“HPV” and “LPEP”), HLA A2 (“GLC” and “CLG”), HLA A24 (“TYG” and “PYL”), HLA B8 (“FLR” and “RAK”) restricted peptides, or gp350 OLP PepMix™ EBV, a pool of 224 peptides derived from a peptide scan (15mers with 11 aa overlap) through envelope glycoprotein GP350/GP340 (Swiss-Prot ID: P03200) of Epstein-Barr virus (HHV4) (Product Code: PM-EBV-GP350/GP340; JPT Peptide Technologies GmbH, Berlin, Germany). Splenocytes were stimulated in the presence of Golgiplug™ and Golgistop™ for 5 hours in a 37 °C humidified incubator with 6.5% CO_2_. After incubation, cells were washed twice, then incubated with Live/Dead™ near IR (invitrogen, cat# L34976, 1:250), FITC-conjugated anti-CD4 (BD Biosciences, cat# 553651, 1:200), and PerCP-Cy5.5 conjugated anti-CD8 (BD Biosciences, cat# 551162, 1:200). Cells were fixed and permeabilized using a BD Cytofix/Cytoperm™ kit, then incubated with PE-conjugated anti-IFNγ (BD Biosciences, cat# 554412, 1:200), PE-Cy7 conjugated anti-TNFα (BD Biosciences, cat# 557844, 1:200), and APC-conjugated anti-IL-2 PE (BD Biosciences, cat# 554429, 1:10). Cells were acquired on a BD FACSCanto™ II and data was analyzed (Supplementary Fig. S13) using FlowJo™ software.

### Expanded ICS assay

Splenocytes (7 × 10^6^) were stimulated with 1 μg/mL of HLA B35 (“HPV” and “LPEP”), HLA A2 (“GLC” and “CLG”), HLA A24 (“TYG” and “PYL”) and HLA B8 (“FLR” and “RAK”) restricted peptides or with 1 μg/mL of gp350 OLP PepMix™. Cells were cultured in a 24-well plate for 10 days at 37 °C, 10% CO_2_, and cultures were supplemented with IL-2 on days 2, 5, and 8. On day 10, the expanded T cells were stimulated with respective epitope peptides and then T cell specificity and polyfunctionality were assessed using multiparametric ICS assay, as described above.

### EBV lymphoma model

EBVpoly-stimulated T cells were expanded from a healthy EBV seropositive donor (HLA A*01:01, A*02:01, B*44:02) as outlined above. Serum-containing gp350-specific antibodies were pooled from HLA B35 transgenic mice immunized with EBV vaccine. Three groups of NOD.Rag1KO.IL2RγcKO (NRG) mice (6 to 8 weeks old) were either Mock treated with PBS (100 µL i.v.; Group 1; *n* = 6), or administered EBVpoly-stimulated T cells (2×10^7^/mouse, i.v.) in combination with mouse serum containing gp350-specific antibodies (100 µL/mouse, i.p.; Group 2; *n* = 6) or EBVpoly-stimulated T cells alone (2 × 10^7^/mouse, i.v.; Group 3; *n* = 6) on day −1. On day 0, mice were injected with HLA-matched (HLA A*02:01, B*44:02) EBV-transformed lymphoblastoid cells (LCLs, generated in-house) (4 × 10^6^ cells/mouse, s.c.) into the right flank. On day 7 after LCL engraftment, mice were retreated as outlined for day −1. Tumor growth was monitored every 2–3 days using Vernier callipers and tumor volume calculated as tumor length (mm) × tumor width × (tumor width × 0.5). Volumes exceeding 4 mm^3^ were reported. Mice were killed when tumor volume reached 500 mm^3^, tumor became necrotic, or when mice lost more than 20% of their body weight. On the day of killing frequencies of human B cells and EBV-specific CD8^+^ T cells were assessed in splenocytes and blood using flow cytometry analysis. For human EBV B cell analysis, splenocytes and blood lymphocytes were stained with Live/Dead™ near IR (Invitrogen, cat# L34976, 1:250), APC-Fire™ 750-conjugated anti-mouse CD45 (BioLegend cat# 147714, 1:100), V500-conjugated anti-human CD45 (Becton Dickinson cat# 560777, 1:25), PE-Cy5-conjugated anti-human CD19 (Becton Dickinson cat# 555414, 1:25), and BV711-conjugated anti-human CD3 (BioLegend cat# 344838, 1:100). For assessment of EBV-specific CD8^+^ T cells, splenocytes and blood lymphocytes were stimulated with HLA B44-restricted (EEN, EEC, and VEI) and HLA A2-restricted (GLC and CLG) peptide epitopes (0.2 μg/mL) in the presence of Golgiplug™ and Golgistop™ for 4 hours in a 37 °C humidified incubator with 6.5% CO_2_. Following incubation, these cells were stained with Live/Dead™ near IR (Invitrogen, cat# L34976), APC-Fire™ 750-conjugated anti-mouse CD45 (BioLegend cat# 147714, 1:100), V500-conjugated anti-human CD45 (Becton Dickinson cat# 560777, 1:25), PE-Cy5-conjugated anti-human CD19 (Becton Dickinson cat# 555414, 1:25), BV711-conjugated anti-human CD3 (BioLegend cat# 344838, 1:100), Pacific Blue-conjugated anti-human CD4 (Becton Dickinson cat# 558116, 1:200), PerCP-Cy5.5-conjugated anti-human CD8 (Invitrogen cat# 45-0088-42, 1:200), FITC-conjugated anti-human CD107a (Becton Dickinson cat# 555800, 1:10), APC-conjugated anti-human TNF (Becton Dickinson cat# 554514, 1:200), AF700-conjugated anti-human IFNγ (Becton Dickinson cat# 557995, 1:50), and PE-conjugated anti-human IL-2 (Invitrogen cat# 12-7029-42, 1:50). Cells were acquired on a BD LSRFortessa™ and data was analyzed (Supplementary Fig. S14) using FlowJo™ software.

### Statistics and reproducibility

Generally, sample sizes of *n* = 6–10 animals for treatment groups and *n* = 4–6 animals for control groups were used; no statistical method was used to predetermine sample size. No data were excluded from the analyses. Age and litter-matched animals were randomly assigned to treatment or control groups. Treatment and control groups of mice were blinded with group numbers. Investigators were not aware of treatment or control groups. For comparing two experimental groups, the two-sided Mann–Whitney test was used. For all analyses involving three or more groups, one-way ANOVA was performed with subsequent Tukey, Dunnett or Šidák post-hoc analysis, as appropriate. Statistical analysis was performed using Graphpad Prism v9.4.

### Reporting summary

Further information on research design is available in the Nature Portfolio Reporting Summary linked to this article.

## Supplementary information

Supplementary Information

Reporting Summary

## Data Availability

Data generated and analysed during this study are included in this published article including its supplementary information files. Source data are provided with this paper in associated Source Data files. Graphics are available through Figshare (10.6084/m9.figshare.23501655). Any additional datasets generated during and/or analysed during the current study are available from the corresponding author on reasonable request. Source data are provided with this paper.

## Source data

Source Data
