# Supplementary material for: Lymph node targeted multi-epitope subunit vaccine promotes effective immunity to EBV in HLA-expressing mice
Source: Nat Commun. 2023 Aug 8;14:4371. doi: 10.1038/s41467-023-39770-1 (PMC10409721; doi:10.1038/s41467-023-39770-1)
Supplement: Supplementary file 1 — Supplementary Information [file 41467_2023_39770_MOESM1_ESM.pdf]

Supplementary Materials for

Lymph node targeted multi-epitope subunit vaccine promotes effective immunity to EBV in HLA-expressing mice

Vijayendra Dasari[1#], Lisa K. McNeil[2], Kirrilee Beckett[1], Matthew Solomon[1], George Ambalathingal[1], Thuy T Le[1], Archana Panikkar[1], Caitlyn Smith[1], Martin P. Steinbuck[2], Aniela Jakubowski[2], Lochana M. Seenappa[2], Erica Palmer[2], Jeff Zhang[2], Christopher M. Haqq[2], Peter C. DeMuth[2,3] and Rajiv Khanna[1,3,#]

[1] QIMR Centre for Immunotherapy and Vaccine Development, Tumour Immunology Laboratory, Infection and Inflammation Program, Berghofer Medical Research Institute, Brisbane, Australia

[2] Elicio Therapeutics, Inc. Boston, MA USA

[3] authors contributed equally

[#]Corresponding author

Prof. Rajiv Khanna or Dr. Vijayendra Dasari, QIMR Berghofer Medical Research Institute, 300 Herston Road, Herston (Qld) Australia; Email: rajiv.khanna@qimr.edu.au or vijayendra.dasari@qimr.edu.au

Tel: 61-7-3362 0385

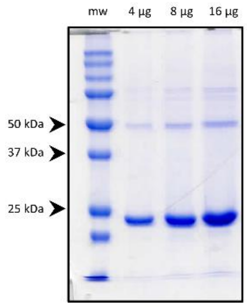

Supplemental Figure S1: Expression and purification of EBVpoly.

Representative SDS-PAGE gel of purified EBVpoly protein. Predicted size was 25.9 kDa. Data are representative of one experiment. Source data are provided as a Source Data file.

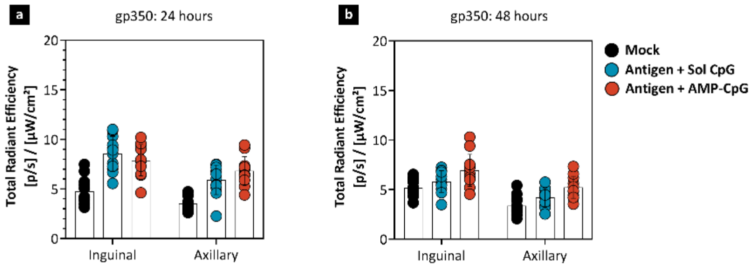

Supplemental Figure S2: gp350 accumulation in lymph nodes.

C57Bl/6J mice were immunized with 8 µg EBVpoly-AF594 and 10 µg gp350-AF647 admixed with 1.2 nmol soluble- or AMP-CpG. **a-b** Quantification of total radiant efficiency in inguinal and axillary lymph nodes analyzed *ex vivo* by IVIS at **a** 24 and **b** 48 hours post primer dose. Mock treatments represent fluorescence-negative controls collected from mice receiving equivalent amounts of AMP-CpG and unlabeled antigens. Data are representative of one experiment. Data are representative of one experiment with n = 12 per group. Values depicted are mean ± standard deviation. If statistics are not indicated, then the comparison was not significant. Source data are provided as a Source Data file.

	Inguinal: 24 hours			Axillary: 24 hours		
	Mock	Sol	AMP	Mock	Sol	AMP
**G-CSF**	37.5	140.7	207.0	3.5	15.3	34.3
**GM-CSF**	14.3	70.0	55.6	8.4	21.6	23.4
**M-CSF**	19.7	38.9	43.8	11.5	20.3	23.3
**IFNg**	9.9	3913.7	3487.8	6.6	696.1	1272.4
**TNFa**	9.4	59.0	73.1	6.7	22.3	35.1
**IL-2**	8.8	8.0	9.1	8.7	7.5	7.1
**IL-12p40**	6.7	11.2	14.2	8.1	12.3	10.5
**IL-6**	9.7	598.3	562.3	7.2	112.9	195.7
**IL-4**	1.4	0.9	1.0	1.2	0.5	0.5
**IL-10**	16.7	26.9	32.0	20.9	20.3	16.3
**IP-10**	156.3	5305.5	4684.2	135.0	2535.5	3590.8
**KC**	22.1	192.8	202.0	13.9	42.9	61.6
**MCP-1**	68.8	2150.3	1952.5	28.7	910.7	1334.4
**MIP1a**	62.4	628.9	714.0	47.4	256.5	440.7
**MIP1b**	65.0	1238.7	1467.3	37.7	544.1	928.5
**MIG**	2636.8	10000.0	10000.0	1354.7	7115.2	5529.8
**IL-1b**	10.5	74.6	88.1	9.1	19.2	29.0

	Inguinal: 48 hours			Axillary: 48 hours		
	Mock	Sol	AMP	Mock	Sol	AMP
**G-CSF**	25.6	18.6	52.3	2.0	1.5	3.3
**GM-CSF**	2.5	16.5	19.8	3.0	6.1	10.1
**M-CSF**	8.5	20.6	25.2	7.1	11.0	12.7
**IFNg**	4.0	146.2	1106.6	4.4	22.4	194.5
**TNFa**	3.0	12.3	27.9	2.8	3.3	9.8
**IL-2**	5.2	6.9	7.3	6.2	5.7	5.5
**IL-12p40**	3.2	6.2	8.8	2.8	3.5	6.5
**IL-6**	4.7	50.8	201.6	4.8	10.3	46.4
**IL-4**	1.3	0.3	0.4	1.0	1.5	1.3
**IL-10**	5.9	15.1	18.0	12.2	13.1	19.1
**IP-10**	65.8	637.0	1334.4	115.1	328.8	960.8
**KC**	15.5	24.0	40.8	12.2	12.0	19.4
**MCP-1**	25.4	213.7	793.6	10.0	40.3	310.3
**MIP1a**	3.2	117.4	311.3	13.9	33.1	132.2
**MIP1b**	3.2	283.6	670.5	10.6	101.6	317.0
**MIG**	1389.8	4165.6	3479.3	1154.9	1900.7	2292.5
**IL-1b**	2.9	15.7	29.2	3.6	4.6	10.2

Supplemental Figure S3: AMP-CpG enhances delivery of EBVpoly to the lymph node alongside comprehensive immune activation.

C57Bl/6J mice were immunized with 8 µg EBVpoly-AF594 and 10 µg gp350-AF647 admixed with 1.2 nmol soluble- or AMP-CpG (n = 6 mice per group, 2 lymph nodes per mouse). Quantification of cytokine concentrations in lymph nodes by Luminex. Listed are protein analyte concentrations (pg/mL) in lymph nodes. Data are representative of one experiment.

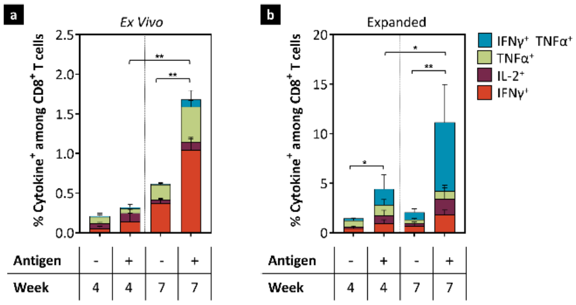

Supplemental Figure S4: Vaccination with AMP-CpG induces CD8⁺ T cell responses to gp350.

HLA-B*35:01 transgenic mice (n = 6 per vaccine group and n = 4 per control group) were immunized on Weeks 0, 3 and 6 with 40 µg EBVpoly and 10 µg gp350 proteins admixed with 1.2 nmol AMP-CpG and T cell responses were analyzed on Week 4 and Week 7. The control group was immunized with AMP-CpG alone. Splenocytes were stimulated with gp350 OLPs **a** *ex vivo* or **b** after expansion with gp350 OLPs in an ICS assay. Shown are frequencies of IFNγ, IL-2, TNFα and IFNγ TNFα double positive $CD8^+$ T cells. Data are representative of one experiment. Values depicted are mean ± standard deviation. * $p < 0.05$; ** $p < 0.01$ by two-sided Mann-Whitney test applied to cytokine+ T cell frequencies. If statistics are not indicated, then the comparison was not significant. The exact p-values are as follows. **Panel a**: AMP vaccine at Week 7 to AMP vaccine at Week 4-0.0022, and AMP vaccine to AMP-CpG at Week 7-0.0095. **Panel b**: AMP vaccine to AMP CpG at week 4-0.0381, AMP vaccine at Week 7 to AMP vaccine at Week 4-0.0260, and AMP vaccine to AMP-CpG at Week 7-0.0095. Source data are provided as a Source Data file.

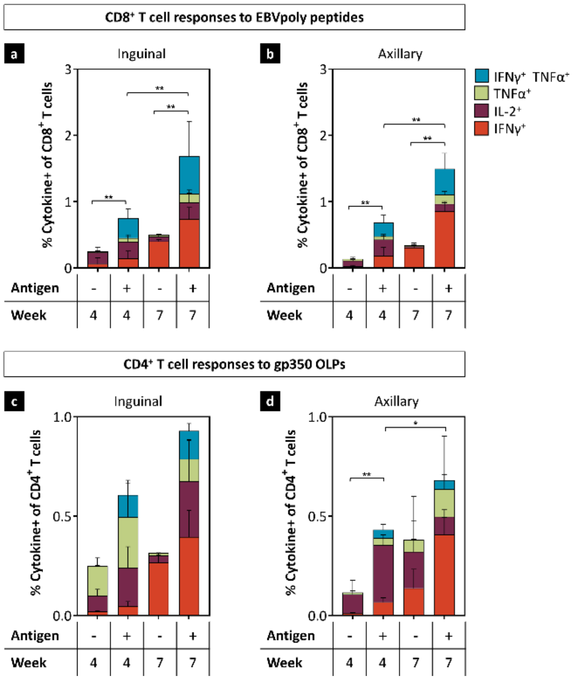

Supplemental Figure S5: AMP-CpG vaccination induces T cell responses in lymph nodes.

HLA-B*35:01 transgenic mice (n = 9 per vaccine group and n = 5 per control group) were immunized on Weeks 0, 3 and 6 with 40 µg EBVpoly and 10 µg gp350 proteins admixed with 1.2 nmol AMP-CpG and T cell responses were analyzed on Week 4 and 7. Lymph node cells were stimulated in an ICS assay with EBV CD8$^+$ T cell peptides **a, b** or gp350 OLPs **c, d**. The control group was immunized with AMP-CpG alone. **a** CD8$^+$ T cells, Inguinal lymph node, **b** CD8$^+$ T cells, Axillary lymph node, **c** CD4$^+$ T cells, Inguinal lymph node and **d** CD4$^+$ T cells, Axillary lymph node. Shown are frequencies of IFNγ, IL-2, TNFα and IFNγ TNFα double positive CD8$^+$ and CD4$^+$ T cells. Data are representative of one experiment. Values depicted are mean ± standard deviation. * $p < 0.05$; ** $p < 0.01$ by two-sided Mann-Whitney test applied to cytokine+ T cell frequencies. If statistics are not indicated, then the comparison was not significant. The exact p-values are as follows. **Panel a**: AMP vaccine to AMP-CpG at week 4-0.0095, AMP vaccine at Week 7 to AMP vaccine at Week 4-0.0260, and AMP vaccine to AMP-CpG at Week 7-0.0095. **Panel b**: AMP vaccine to AMP-CpG at week 4-0.0381, AMP vaccine at Week 7 to AMP vaccine at Week 4-0.0022, and AMP vaccine to AMP-CpG at Week 7-0.0095. **Panel d**: AMP vaccine to AMP-CpG at week 4-0.0095, AMP vaccine at Week 7 to AMP vaccine at Week 4-0.0152. Source data are provided as a Source Data file.

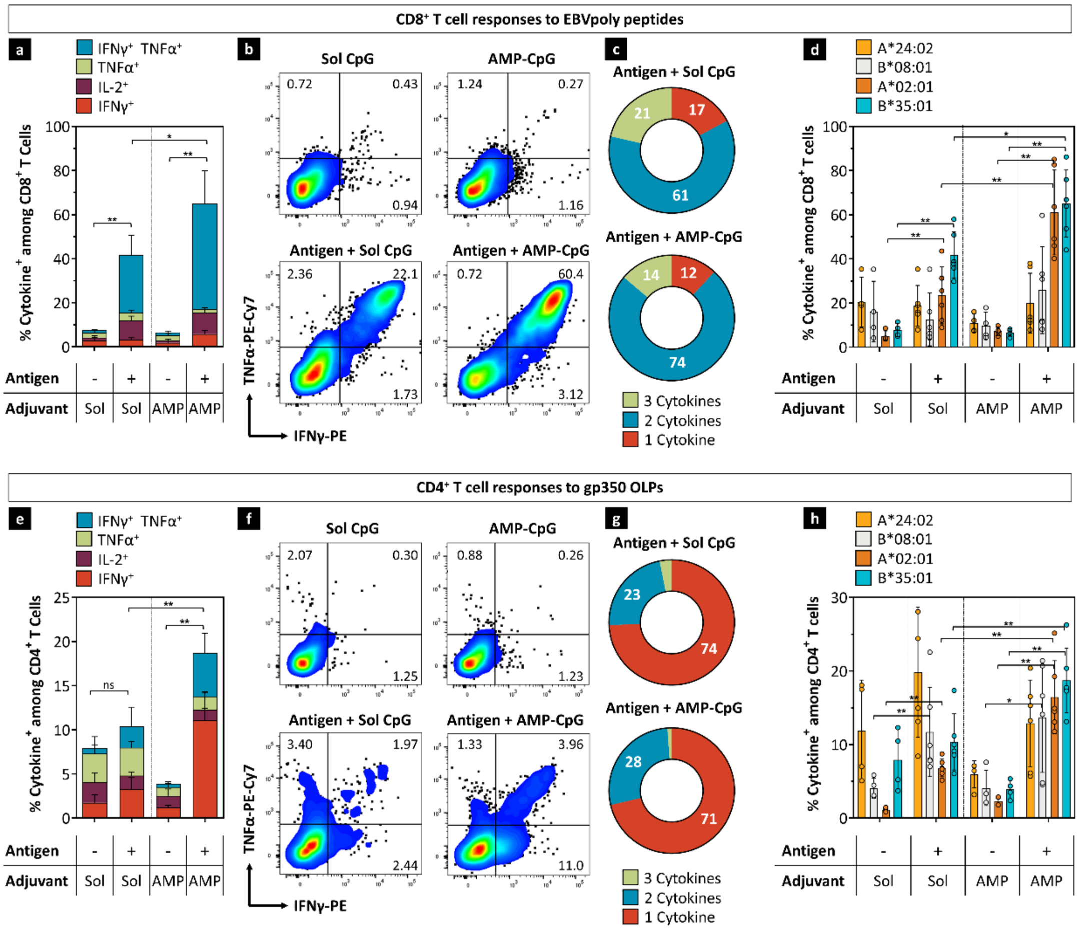

Figure S6: Vaccination with AMP-CpG induces robust polyfunctional EBV-specific T cell responses in expanded splenocytes.

a-h HLA-B*35:01 transgenic mice (n = 6 per vaccine group and n = 4 per control group) were immunized on Weeks 0, 3 and 6 with 40 µg EBVpoly and 10 µg gp350 proteins admixed with 1.2 nmol soluble- or AMP-CpG and T cell responses were analyzed on Week 7. Control groups were immunized with soluble-CpG or AMP-CpG alone. Splenocytes were collected on Week 7 and stimulated with IL-2 and **a-d** EBV CD8$^+$ T cell peptides or **e-h** gp350 OLPs for 10 days. Expanded splenoctyes were restimulated with **a-d** CD8$^+$ T cell peptides and **b-h** gp350 OLPs in an ICS assay. **a, e** Shown are frequencies of IFNγ, IL-2, TNFα and IFNγ TNFα double positive CD8$^+$ or CD4$^+$ T cells, with **b, f** corresponding representative dot plots. **c, g** Pie chart representations of the functional T cell profiles. Pies represent the capacity of T cells to secrete any (1, 2 or 3) of the three cytokines IFNγ, TNFα and IL-2. **d, h** Frequencies of cytokine$^+$ **d** CD8$^+$ T cells and **h** CD4$^+$ T cells in splenocytes to indicated HLA transgenic mice. Data are representative of one experiment. Values depicted are mean ± standard deviation. * $p < 0.05$; ** $p < 0.01$ by two-sided Mann-Whitney test applied to cytokine+ T cell frequencies. If statistics are not indicated, then the comparison was not significant. The exact *p* values are as follows. **Panel a**: Soluble vaccine to soluble CpG 0.0095, AMP vaccine to AMP-

CpG 0.0095 and AMP vaccine to soluble vaccine 0.015. **Panel d**: Soluble vaccine to Soluble CpG for A*24:01-0.761, A*08:01-0.761, A*02:01-0.0095 and B*35:01-0.0095. AMP vaccine to AMP-CpG for A*24:01-0.257, A*08:01-0.114, A*02:01-0.0095 and B*35:01-0.0095. AMP vaccine to Soluble vaccine for A*24:01-0.699, A*08:01-0.179, A*02:01-0.004 and B*35:01-0.015. **Panel e**: Soluble vaccine to soluble CpG 0.609, AMP vaccine to AMP-CpG 0.0095 and AMP vaccine to soluble vaccine 0.015. **Panel h**: Soluble vaccine to Soluble CpG for A*24:01-0.257, A*08:01-0.0095, A*02:01-0.0095 and B*35:01-0.609. AMP vaccine to AMP-CpG for A*24:01-0.257, A*08:01-0.038, A*02:01-0.0095 and B*35:01-0.0095. AMP vaccine to Soluble vaccine for A*24:01-0.309, A*08:01-0.937, A*02:01-0.002 and B*35:01-0.008. Source data are provided as a Source Data file.

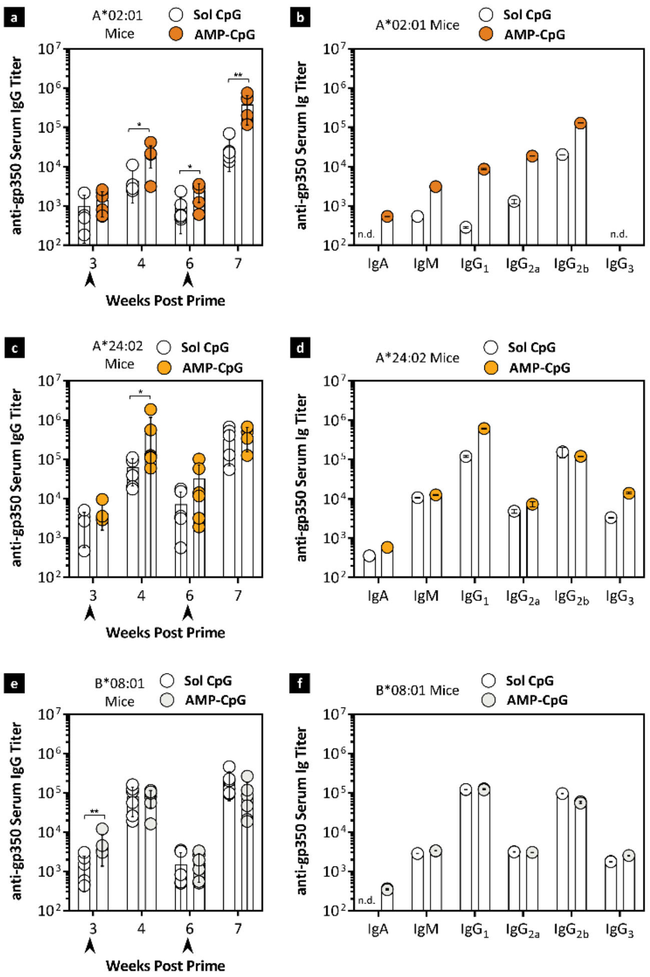

Supplemental Figure S7: Vaccination with AMP-CpG induces gp350-specific antibody responses in multiple HLA transgenic mice.

HLA-A*02:01, A*24:02 and B*08:02 transgenic mice (n = 6 per vaccine group and n = 4 per control group) were immunized on Weeks 0, 3 and 6 with 40 µg EBVpoly and 10 µg gp350 proteins admixed with 1.2 nmol AMP-CpG and serum Ig titers to gp350 were assessed by ELISA. The control group was immunized with AMP-CpG alone. Longitudinal IgG titers for **a** A*02:01, **c** A*24:02 and **e** B*08:01 transgenic mice and serum Ig subtype titers at Week 7 for **b** A*02:01, **d** A*24:02 and **f** B*08:01 transgenic mice. Data are representative of one experiment. Values depicted are mean ± standard deviation. Arrows indicate immunization days. * $p < 0.05$; ** $p < 0.01$ by two-sided Mann-Whitney test. If statistics are not indicated, then the comparison was not significant. Exact *p* values are as follows: **Panel a:** Soluble vaccine to AMP vaccine at week 3-0.24 , week 4-0.026 (week 4), week 60.015 and week 7-0.002. **Panel c**: Soluble vaccine to AMP vaccine at week 3-0.3939, week 4 0.026 (week 4, week 6-0.4848 and week 7-0.785. **Panel f**: Soluble vaccine to AMP vaccine at week 3-0.002 (week 3, week 4-0.069, week 6-0.818and week 7-0.093. Source data are provided as a Source Data file.

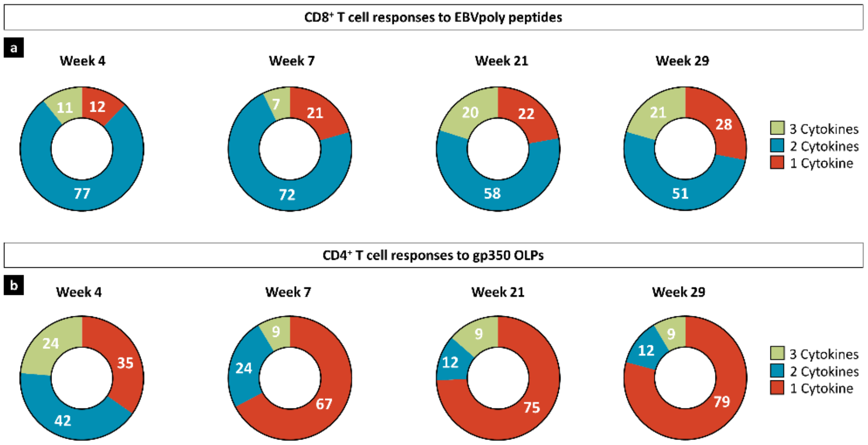

Supplemental Figure S8: Longitudinal polyfunctional T cell response to AMP-CpG immunization. HLA-B*35:01 transgenic mice (n = 9 per vaccine group and n = 5 per control group) were immunized on Weeks 0, 3 and 6 with 40 µg EBVpoly and 10 µg gp350 proteins admixed with 1.2 nmol AMP-CpG. The control group was immunized with AMP-CpG alone. Splenocytes were stimulated with **a** EBV CD8^{+} T cell peptides or **b** gp350 OLPs in an ICS assay. Shown are pie chart representations of the functional T cell profiles. Pies represent the capacity of T cells to secrete any (1, 2 or 3) of the three cytokines IFNγ, IL-2 and TNFα. **a** CD8^{+} T cell response, **b** CD4^{+} T cell response. Data are representative of one experiment. Source data are provided as a Source Data file.

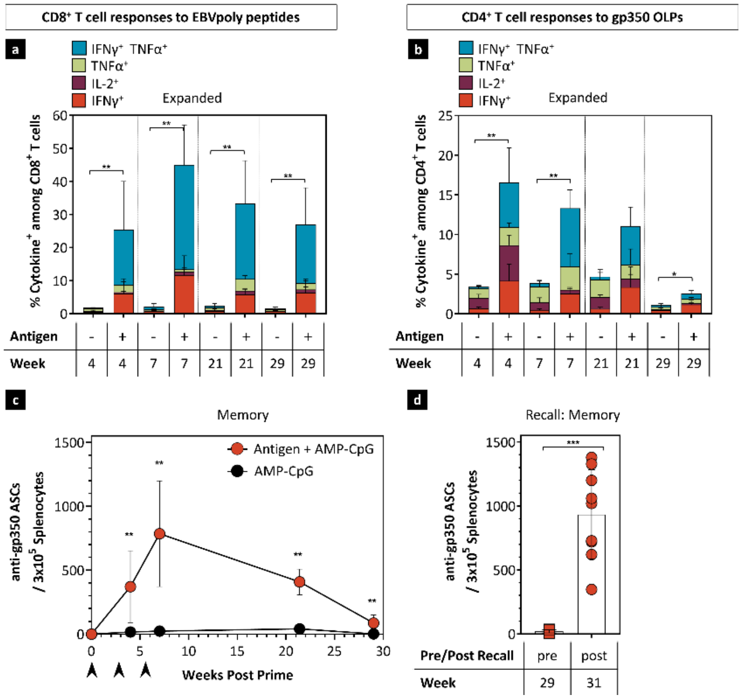

Supplemental Figure S9. Vaccination with AMP-CpG induces durable EBV-specific T cell and humoral responses in expanded memory cells.

a, b HLA-B*35:01 transgenic mice (n = 9 per vaccine group and n = 5 per control group) were immunized on Weeks 0, 3 and 6 with 40 µg EBVpoly and 10 µg gp350 proteins admixed with 1.2 nmol AMP-CpG and T cell and humoral responses were analyzed at long term timepoints. The control group was immunized with AMP-CpG alone. Shown are *in vitro* stimulated cytokine+ **a** $CD8^+$ or **b** $CD4^+$ T cell frequencies at different timepoints, **c** expanded memory ASC ELISPOT measured frequency of gp350-specific ASCs per $3x10^5$ splenocytes, **d** Week 31 memory ASC recall response to a recall vaccination at Week 30. Data are representative of one experiment. Values depicted are mean ± standard deviation. Black arrows indicate immunization days. * $p < 0.05$; ** $p < 0.01$ by two-sided Mann-Whitney test applied to cytokine+ T cell frequencies. If statistics are not indicated, then the comparison was not significant. Exact p values are as follows**. Panel a**: AMP vaccine to AMP-CpG control at week 40.0095, week 7-0.0095, week 21-0.0095 and week 29-0.0095. **Panel b**. AMP vaccine to AMP-CpG at week 4-0.0095 , week 7-0.0095 , week 21-0.0667 and week 29-0.0381. **Panel c**: AMP vaccine to AMP-CpG week 4-0.0095 , week 7-0.0095 , week 21 0.0095 and week 29-0.0095. **Panel d**: AMP vaccine week 29 to 31-0.0004. Source data are provided as a Source Data file.

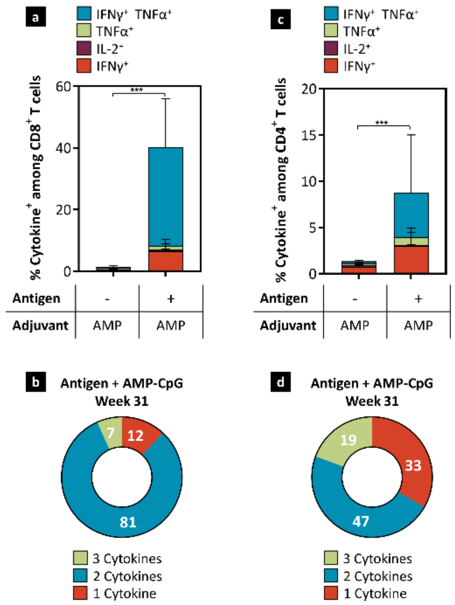

Supplemental Figure S10: Recall polyfunctional T cell response to AMP-CpG immunization.

HLA-B*35:01 transgenic mice (n = 9 per vaccine group and n = 5 per control group) were immunized on Weeks 0, 3 and 6 and 30 with 40 µg EBVpoly and 10 µg gp350 proteins admixed with 1.2 nmol AMP-CpG. The control group was immunized with AMP-CpG alone. Expanded splenoctyes from recall Week 31 were stimulated with EBV CD8+ T cell peptides or gp350 OLPs in an ICS assay. Shown are frequencies of IFNγ, IL-2, TNFα and IFNγ TNFα double positive cells from **a** expanded CD8+ T cells and **c** expanded CD4+ T cells. Pie chart representations of the functional T cell profile are shown for **b** *ex vivo* CD8+ T cells and **d** *ex vivo* CD4+ T cells. Data are representative of one experiment. Values depicted are mean ± standard deviation. * $p < 0.05$; ** $p < 0.01$ by two-sided Mann-Whitney test applied to cytokine+ T cell frequencies. If statistics are not indicated, then the comparison was not significant. Exact p values are as follows: **Panel a**: AMP vaccine to AMP-CpG-0.001. **Panel b**: AMP vaccine AMP-CpG 0.0010. Source data are provided as a Source Data file.

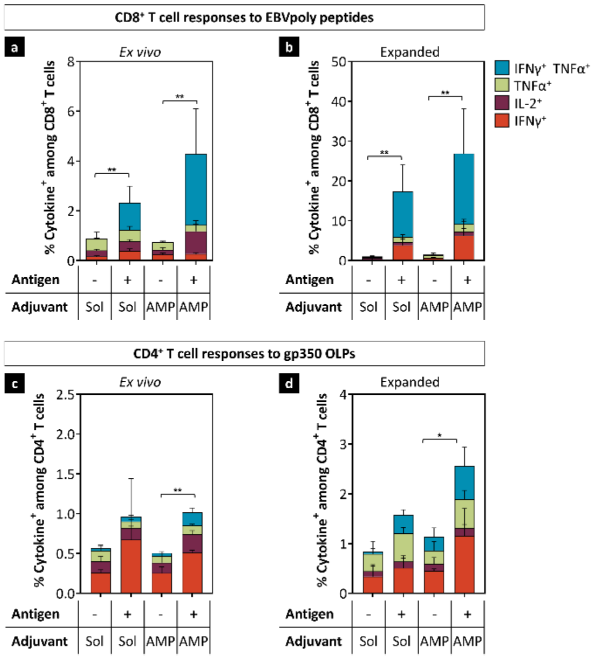

Supplemental Figure S11: Comparison of T cell responses at Week 29 for soluble CpG and AMP-CpG immunized mice.

HLA-B*35:01 transgenic mice (n = 9 per vaccine group and n = 6 per control group) were immunized on Weeks 0, 3 and 6 with 40 µg EBVpoly and 10 µg gp350 proteins admixed with 1.2 nmol soluble- or AMP-CpG and T cell responses were analyzed on Week 29. Control groups were immunized with soluble-CpG or AMP-CpG alone. *Ex vivo* or expanded splenocytes were stimulated with EBV CD8$^+$ T cell peptides **a, b** or gp350 OLPs **c, d** in an ICS assay. Shown are frequencies of IFNγ, IL-2, TNFα and IFNγ TNFα double positive **a** *ex vivo* CD8$^+$ T cells, **b** expanded CD8$^+$ T cells, **c** *ex vivo* CD4$^+$ T cells and **d** expanded CD4$^+$ T cells. Data are representative of one experiment. Values depicted are mean ± standard deviation. * $p < 0.05$; ** $p < 0.01$ by two-sided Mann-Whitney test applied to cytokine+ T cell frequencies. If statistics are not indicated, then the comparison was not significant. Exact *p* values are as follows: **Panel a**: A Soluble CpG vaccine to vs Soluble CpG- 0.0095, AMP vaccine to AMP-CpG-0.0095 and Soluble vaccine to AMP vaccine-0.0649. **Panel b**: Soluble vaccine to vs Soluble CpG-0.0095, AMP vaccine vs AMP-CpG-0.0095 and Soluble Vaccine to AMP vaccine-0.3939. **Panel c**: Soluble vaccine vs soluble CpG-0.2571, AMP vaccine to AMP-CpG-0.0095 and Soluble vaccine to AMP-CpG vaccine- 0.0649. **Panel d:** Soluble vaccine to Soluble CpG-0.114, AMP-CpG vaccine to AMP-CpG- 0.038 and Soluble vaccine vs AMPvaccine-0.093. Source data are provided as a Source Data file.

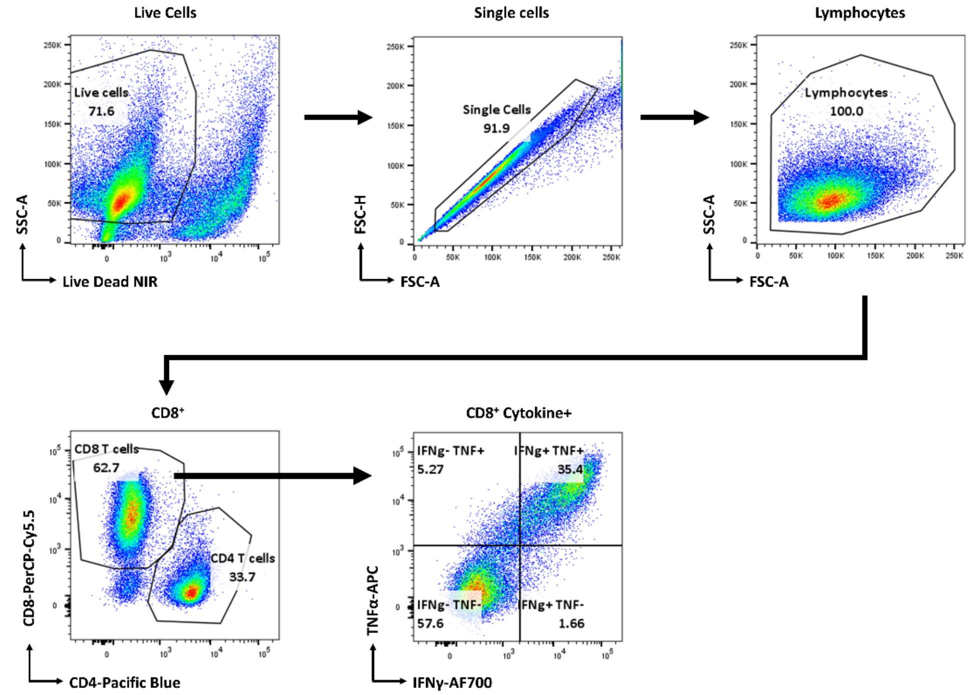

Supplemental Figure S12: Gating strategy of Expanded PBMCs from healthy EBV seropositive patients.

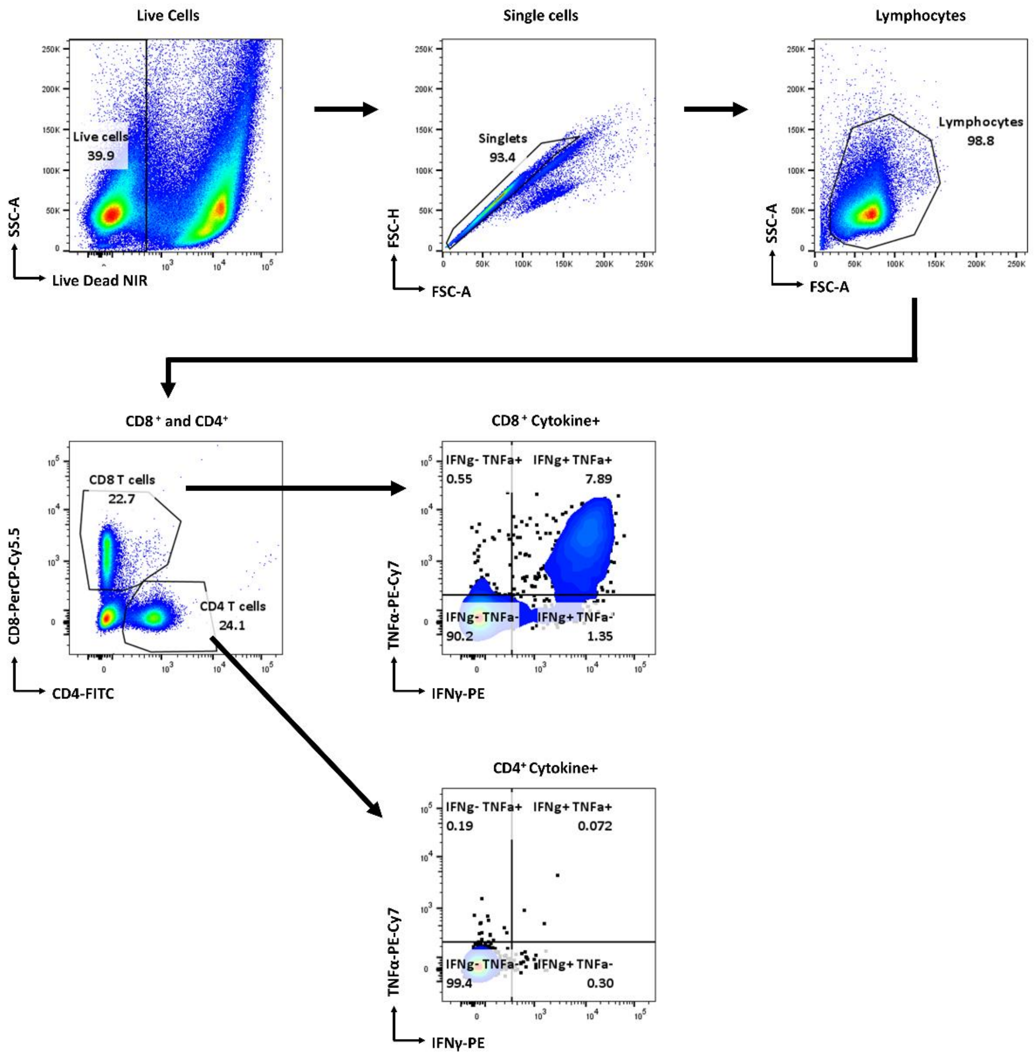

Supplemental Figure S13: Gating strategy of EBV-specific T cell responses in splenocytes.

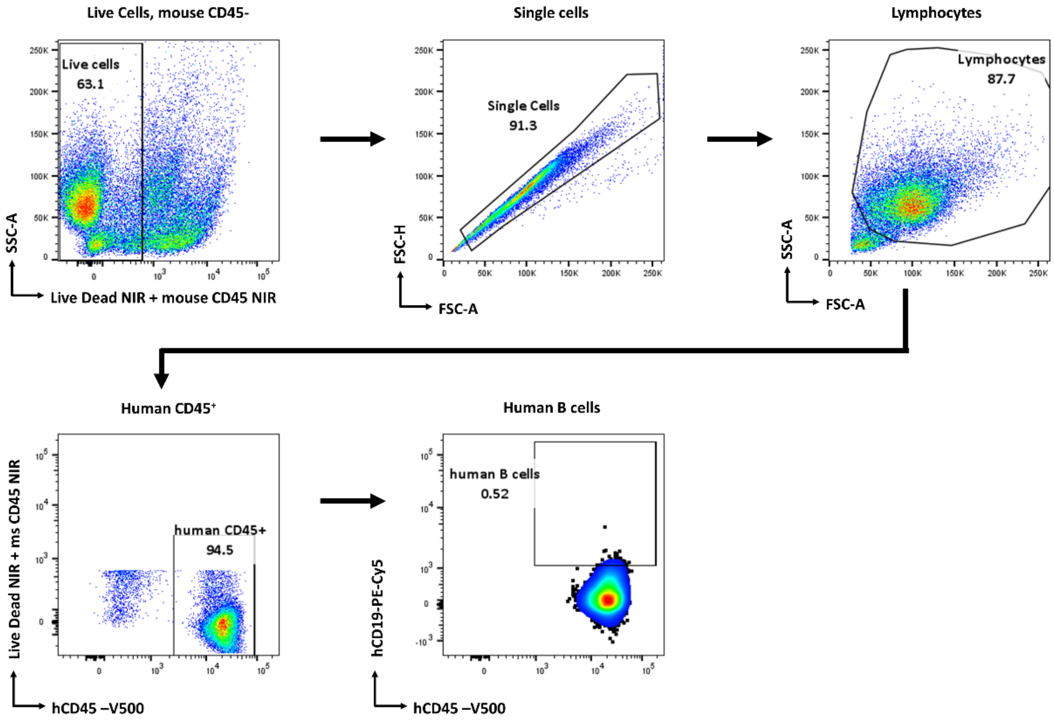

Supplemental Figure S14: Gating strategy of human B cells in splenocytes and blood of NRG mice.
