# Supplementary material for: Lymph node targeted multi-epitope subunit vaccine promotes effective immunity to EBV in HLA-expressing mice
Source: Nat Commun. 2023 Aug 8;14:4371. doi: 10.1038/s41467-023-39770-1 (PMC10409721; doi:10.1038/s41467-023-39770-1)
Supplement: Supplementary file 2 — Reporting Summary [file 41467_2023_39770_MOESM2_ESM.pdf]

nature portfolio

Corresponding author(s): Rajiv Khanna

Last updated by author(s): Jun 9, 2023

Reporting Summary

Nature Portfolio wishes to improve the reproducibility of the work that we publish. This form provides structure for consistency and transparency in reporting. For further information on Nature Portfolio policies, see our Editorial Policies and the Editorial Policy Checklist.

Statistics

For all statistical analyses, confirm that the following items are present in the figure legend, table legend, main text, or Methods section.

n/a	Confirmed	
☐	☒	The exact sample size (*n*) for each experimental group/condition, given as a discrete number and unit of measurement
☐	☒	A statement on whether measurements were taken from distinct samples or whether the same sample was measured repeatedly
☐	☒	The statistical test(s) used AND whether they are one- or two-sided<br>*Only common tests should be described solely by name; describe more complex techniques in the Methods section.*
☐	☒	A description of all covariates tested
☐	☒	A description of any assumptions or corrections, such as tests of normality and adjustment for multiple comparisons
☐	☒	A full description of the statistical parameters including central tendency (e.g. means) or other basic estimates (e.g. regression coefficient) AND variation (e.g. standard deviation) or associated estimates of uncertainty (e.g. confidence intervals)
☐	☒	For null hypothesis testing, the test statistic (e.g. *F*, *t*, *r*) with confidence intervals, effect sizes, degrees of freedom and *P* value noted<br>*Give P values as exact values whenever suitable.*
☒	☐	For Bayesian analysis, information on the choice of priors and Markov chain Monte Carlo settings
☒	☐	For hierarchical and complex designs, identification of the appropriate level for tests and full reporting of outcomes
☒	☐	Estimates of effect sizes (e.g. Cohen's *d*, Pearson's *r*), indicating how they were calculated

Our web collection on statistics for biologists contains articles on many of the points above.

Software and code

Policy information about availability of computer code

Data collection	IVIS Spectrum In Vivo Imaging System<br>BD LSR FortessaTM<br>BD FACSCanto IITM<br>AID ELISpot Reader
Data analysis	The Immune Epitope Database (IEDB, v 2.27) population coverage of CD8+ T-cell epitope-based analysis was used to calculate the worldwide coverage. IEDB population coverage resource is a freely available resource funded by NIAID. It catalogs experimental data on antibody and T cell epitopes studied in humans, non-human primates, and other animal species in the context of infectious disease, allergy, autoimmunity and transplantation. http://tools.iedb.org/population/<br>FlowJo TM V10<br>GraphPad Prism v9.4<br>Living Image v4.7.3

For manuscripts utilizing custom algorithms or software that are central to the research but not yet described in published literature, software must be made available to editors and reviewers. We strongly encourage code deposition in a community repository (e.g. GitHub). See the Nature Portfolio guidelines for submitting code & software for further information.

Data

Policy information about availability of data

All manuscripts must include a data availability statement. This statement should provide the following information, where applicable:

- Accession codes, unique identifiers, or web links for publicly available datasets
- A description of any restrictions on data availability
- For clinical datasets or third party data, please ensure that the statement adheres to our policy

We have uploaded all raw data in Excel format along with manuscript as a Source Data file.

Research involving human participants, their data, or biological material

Policy information about studies with human participants or human data. See also policy information about sex, gender (identity/presentation), and sexual orientation and race, ethnicity and racism.

Reporting on sex and gender	Sex and gender based analysis is not relevant for the study. Protected health information was not collected as recommended by the ethical review committee for the purposes of this translational work.
Reporting on race, ethnicity, or other socially relevant groupings	Analysis of race, ethnicity, and other social groupings are not relevant for the study. Protected health information was not collected as recommended by the ethical review committee for the purposes of this translational work.
Population characteristics	Healthy volunteers who were willing to offer blood were recruited. No additional information was collected as requested by the ethical review committee for the purposes of this translational work.
Recruitment	All healthy volunteers recruited in the study offered blood samples and gave written informed consent. No self-selection bias was involved in this study. The only criteria used for recruitment was EBV seropositivity.
Ethics oversight	The study was approved by QIMR Berghofer Medical Research Ethics Committee under project number P2282. We have provided ethics statement in Materials and methods section.

Note that full information on the approval of the study protocol must also be provided in the manuscript.

Field-specific reporting

Please select the one below that is the best fit for your research. If you are not sure, read the appropriate sections before making your selection.

[x] Life sciences [ ] Behavioural & social sciences [ ] Ecological, evolutionary & environmental sciences

For a reference copy of the document with all sections, see nature.com/documents/nr-reporting-summary-flat.pdf

Life sciences study design

All studies must disclose on these points even when the disclosure is negative.

Sample size	Any statistical difference p < 0.05 between control group and test group is considered as real vaccine response. Assuming a minimum sample size of 6 mice and 80% power and a two-sided alpha of 0.05, we will be able to detect an effect size of 1.75 between two different groups, unadjusted for any multiple comparisons. This is a very large difference between test and control groups.
Data exclusions	No data were excluded from the analyses.
Replication	We evaluated EBV vaccine immunogenicity in various HLA transgenic mice in multiple experiments and we observed reproducible results. Results presented were from individual groups of HLA-expressing mice. Collective results from short term and long term experiments produced reproducible data.
Randomization	Age matched female HLA transgenic mice from different litters were pooled and randomized to allocate into different experimental groups before the experiment. Treatment and control groups of mice were blinded with group numbers. Investigators were not aware of treatment or control groups.
Blinding	Except for studies assessing lymph node fluorescence and multiplexed cytokine analyses, treatment and control groups of mice were blinded with group numbers. Investigators were not aware of treatment and control groups.

Reporting for specific materials, systems and methods

We require information from authors about some types of materials, experimental systems and methods used in many studies. Here, indicate whether each material, system or method listed is relevant to your study. If you are not sure if a list item applies to your research, read the appropriate section before selecting a response.

Materials & experimental systems

n/a	Involved in the study	
	x	Antibodies
	x	Eukaryotic cell lines
x		Palaeontology and archaeology
	x	Animals and other organisms
x		Clinical data
x		Dual use research of concern
x		Plants

Methods

n/a	Involved in the study	
x		ChIP-seq
	x	Flow cytometry
x		MRI-based neuroimaging

Antibodies

Antibodies used

Antibody / Source / Catalogue number / Dilution
FITC Anti-Human CD107a BD Pharmingen 555800 10:100
Live/Dead near IR Invitrogen L34976 4:1000
Pacific Blue Mouse Anti-Human CD4 (Clone RPA-T4) BD Pharmingen 558116 5:1000
PerCP – Cyonine 5.5 Anti-Human CD8a (Clone RPA-TB) EBioscience 45008842 2.5:1000
Anti-Human-IL2-PE (Clone MQI-17H12) EBioscience 12-7029-42 20:1000
APC Anti-Human TNFα – MAB 11 BioLegend 502912 5:1000
Alexa Flour 700 Mouse Anti-Human IFN-γ (Clone B27) BD Biosciences 557995 20:1000
CellTrace Violet Invitrogen C34557 1:1000
Anti-Human CD3 (Clone SK7) APC BD Biosciences 340440 40:1000
PE Cy5 Anti-Human CD19 BD Pharmingen 555414 40:1000
FITC Rat Anti-Mouse CD4 (Clone H129-19) BD Biosciences 553651 5:1000
PerCP-Cy 5.5 Rat Anti-Mouse CD8a (Clone 53-6.7) BD Biosciences 551162 5:1000
PE RAT Anti-Mouse IFN-γ BD Biosciences 554412 5:1000
PE Cy7 RAT Anti-Mouse TNF (Clone MP6-XT22) BD Biosciences 557844 5:1000
APC Rat Anti-Mouse IL-2 BD Biosciences 554429 10:1000
APC-Fire 750 Anti-Mouse CD45 (Clone I3/2.3) BioLegend 147714 10:1000
V500 Anti-Human CD45 (Clone HI30) BD Biosciences 560777 40:1000
BV711 Anti-Human CD3 (Clone SK7) BioLegend 344838 10:1000
Goat Anti-Mouse Ig HRP SouthernBiotech 1010-05 1:1000
Goat Anti-Mouse IgA-HRP SouthernBiotech 1040-05 1:1000
Goat Anti-Mouse IgM-HRP SouthernBiotech 1020-05 1:1000
Goat Anti-Mouse IgG1-HRP SouthernBiotech 1070-05 1:1000
Goat Anti-Mouse IgG2a-HRP SouthernBiotech 1080-05 1:1000
Goat Anti-Mouse IgG2b-HRP SouthernBiotech 1090-05 1:1000
Goat Anti-Mouse IgG3-HRP SouthernBiotech 1100-05 1:1000
Mouse IgG ELISpot kit Mabtech AB 3825-2A

Validation

https://www.bdbiosciences.com/en-us/products/reagents/flow-cytometry-reagents/research-reagents/single-color-antibodies-ruo/fitc-mouse-anti-human-cd107a.555800
https://www.thermofisher.com/order/catalog/product/L34976?SID=srch-hj-L34976
https://www.bdbiosciences.com/en-us/products/reagents/flow-cytometry-reagents/research-reagents/single-color-antibodies-ruo/pacific-blue-mouse-anti-human-cd4.558116
https://www.thermofisher.com/antibody/product/CD8a-Antibody-clone-RPA-T8-Monoclonal/45-0088-42
https://www.thermofisher.com/antibody/product/IL-2-Antibody-clone-MQ1-17H12-Monoclonal/12-7029-42
https://www.biolegend.com/en-us/products/apc-anti-human-tnf-alpha-antibody-1343
https://www.bdbiosciences.com/en-us/products/reagents/flow-cytometry-reagents/research-reagents/single-color-antibodies-ruo/alexa-fluor-700-mouse-anti-human-ifn.557995
https://www.thermofisher.com/order/catalog/product/C34557?SID=srch-srp-C34557
https://www.bdbiosciences.com/en-us/products/reagents/flow-cytometry-reagents/clinical-discovery-research/single-color-antibodies-ruo-gmp/apc-mouse-anti-human-cd3.340440
https://www.bdbiosciences.com/en-us/products/reagents/flow-cytometry-reagents/research-reagents/single-color-antibodies-ruo/pe-cy-5-mouse-anti-human-cd19.555414
https://www.bdbiosciences.com/en-us/products/reagents/flow-cytometry-reagents/research-reagents/single-color-antibodies-ruo/fitc-rat-anti-mouse-cd4.553651
https://www.bdbiosciences.com/en-us/products/reagents/flow-cytometry-reagents/research-reagents/single-color-antibodies-ruo/percp-cy-5-5-rat-anti-mouse-cd8a.551162
https://www.bdbiosciences.com/en-us/products/reagents/flow-cytometry-reagents/research-reagents/single-color-antibodies-ruo/pe-rat-anti-mouse-ifn.554412
https://www.bdbiosciences.com/en-us/products/reagents/flow-cytometry-reagents/research-reagents/single-color-antibodies-ruo/pe-cy-7-mouse-anti-human-ifn.557844
https://www.bdbiosciences.com/en-us/products/reagents/flow-cytometry-reagents/research-reagents/single-color-antibodies-ruo/

apc-rat-anti-mouse-il-2.554429
https://www.biolegend.com/en-us/products/apc-fire-750-anti-mouse-cd45-antibody-16472
https://www.bdbiosciences.com/en-us/products/reagents/flow-cytometry-reagents/research-reagents/single-color-antibodies-ruo/v500-mouse-anti-human-cd45.560777
https://www.biolegend.com/en-us/products/brilliant-violet-711-anti-human-cd3-antibody-12007
https://www.southernbiotech.com/goat-anti-mouse-ig-human-ads-hrp-1010-05
https://www.southernbiotech.com/goat-anti-mouse-iga-hrp-1040-05
https://www.southernbiotech.com/goat-anti-mouse-igm-human-ads-hrp-1020-05
https://www.southernbiotech.com/goat-anti-mouse-igg1-human-ads-hrp-1070-05
https://www.southernbiotech.com/goat-anti-mouse-igg2a-human-ads-hrp-1080-05
https://www.southernbiotech.com/goat-anti-mouse-igg2b-human-ads-hrp-1090-05
https://www.southernbiotech.com/goat-anti-mouse-igg3-human-ads-hrp-1100-05
https://www.mabtech.com/products/elispot-flex-mouse-igg-hrp-3825-2h-0

Eukaryotic cell lines

Policy information about cell lines and Sex and Gender in Research

Cell line source(s)	We have specified source of human PBMC in the material and methods section.<br>CHO Cells (ATCC, cat#CCL61, not authenticated). LCL cells were generated at QIMR.
Authentication	Cell lines were not authenticated.
Mycoplasma contamination	Yes all the cells were free of mycoplasma contamination
Commonly misidentified lines (See ICLAC register)	No commonly misidentified cell lines were used in the study.

Animals and other research organisms

Policy information about studies involving animals; ARRIVE guidelines recommended for reporting animal research, and Sex and Gender in Research

Laboratory animals

HLA A2, HLA A24, HLA B8, and HLA B35 mice (6-8 weeks old) were obtained from Institut Pasteur (Paris, France). The HLA transgenic mice were generated on C57BL/6 background by fusing HLA α1α2 H chain domains with a mouse α3 domain and covalently linked to human β2 macroglobulin (Boucherma, R. et al. HLA-A*01:03, HLA-A*24:02, HLA-B*08:01, HLA-B*27:05, HLA-B*35:01, HLA-B*44:02, and HLA-C*07:01 monochain transgenic/H-2 class I null mice: novel versatile preclinical models of human T cell responses. J Immunol 191, 583-593, doi:10.4049/jimmunol.1300483 (2013)).
C57Bl/6J - 6-8 weeks old
NRG NOD.Rag1KO.IL2RγcKO - 6 - 8 weeks old
The QIMR animal facility uses Gamma Irradiated feed and bedding. All irradiated feed and bedding is appropriately packaged to ensure the integrity of the contents during storage. Fine aspen bedding is used in Optimice mouse individually ventilated cages (IVC). Optimice mouse IVC cages include a protective filter that prevent the introduction of dust, dander, dirt and potential disease-causing organisms into the cage. Cages and other equipment are cleaned and replaced as often as needed to maintain clean, dry housing for the animals. Mice are fed with rat &mouse pellets and acidified water is provided. Feed and water is dispensed in to sterile containers in a laminar flow cabinet. Animal rooms are maintained at ventilation (min 16 air changes per hour), temperature between 180 and 240 C and relative humidity between 40% and 70%. All rooms are operated with automatic timers to maintain 12 hours : 12 hours light/dark cycle (7:30am to 7:30pm).
The Elicio/CRADL animal facility uses Alpha Dri Bedding, and animals are housed on Individual ventilated racks. Water is provided ad libitum and is acidified in bottles. Enrichment is provided in 2 forms: either wooden blocks, Bed'R nest, innodome, shepherd shack, wooden sticks, or diamond twists. Mice are housed 5 animals per cage, with 12 hours light and 12 hours dark (7:00am-7:00pm); temperature is maintained at 71F +/- 3, with humidity at 70% +/-5%.

Wild animals	No wild animals were used in the study.
Reporting on sex	Although we don't have sex bias, we have used female HLA transgenic mice in the study because male mice tend to fight. Due to aggressive nature of the male mice, they have been excluded from the studies.
Field-collected samples	No field collected samples were used in the study.
Ethics oversight	Ethics approval to conduct animal experiments were obtained from QIMR Berghofer Medical Research Institute Animal Ethics committee under project number P2241 and Charles River Accelerator and Development Lab Protocol 2021-1259.

Note that full information on the approval of the study protocol must also be provided in the manuscript.

Flow Cytometry

Plots

Confirm that:

- [x] The axis labels state the marker and fluorochrome used (e.g. CD4-FITC).
- [x] The axis scales are clearly visible. Include numbers along axes only for bottom left plot of group (a 'group' is an analysis of identical markers).
- [x] All plots are contour plots with outliers or pseudocolor plots.
- [x] A numerical value for number of cells or percentage (with statistics) is provided.

Methodology

Sample preparation	Following immunization, blood, spleen and lymph nodes were collected. Single cell suspensions from spleens and lymph nodes were made by mechanical disintegration.
Instrument	Cells were acquired on a BD LSR Fortessa Cell Analyzer Model number 647794L6
Software	Data was analyzed using FlowJo™ software. Graphs were compiled using GraphPad Prism.
Cell population abundance	No cell sorting was used for the analysis.
Gating strategy	All gating strategies commenced with Live-Dead NIR vs SSC-A gate to exclude non-viable cells, followed by a single cell gate FSC-H vs FSC-A to exclude doublets, then , FSC-A vs SSC-A to define lymphocytes. CD4 vs CD8 gate was next used to separate CD8+ cells (CD8+CD4-) . For ICS analysis cells, IFNg, TNFa and IL2 production was then assessed in CD8+ T cells using boolean gating.

- [x] Tick this box to confirm that a figure exemplifying the gating strategy is provided in the Supplementary Information.
